# Citrate Suppresses Tumor Growth in Multiple Models through Inhibition of Glycolysis, the Tricarboxylic Acid Cycle and the IGF-1R Pathway

**DOI:** 10.1038/s41598-017-04626-4

**Published:** 2017-07-03

**Authors:** Jian-Guo Ren, Pankaj Seth, Huihui Ye, Kun Guo, Jun-ichi Hanai, Zaheed Husain, Vikas P. Sukhatme

**Affiliations:** 1 Divisions of Interdisciplinary Medicine and Biotechnology, Hematology-Oncology and Nephrology, Department of Medicine and the Cancer Research Institute, Harvard Medical School, 330 Brookline Avenue, Boston, MA 02215 USA; 2 Department of Pathology, Beth Israel Deaconess Medical Center (BIDMC) and Harvard Medical School, 330 Brookline Avenue, Boston, MA 02215 USA; 3 Zhongshan Hospital, Fudan University, Shanghai, 200032 China

## Abstract

In this study we have tested the efficacy of citrate therapy in various cancer models. We found that citrate administration inhibited A549 lung cancer growth and additional benefit accrued in combination with cisplatin. Interestingly, citrate regressed Ras-driven lung tumors. Further studies indicated that citrate induced tumor cell differentiation. Additionally, citrate treated tumor samples showed significantly higher infiltrating T-cells and increased blood levels of numerous cytokines. Moreover, we found that citrate inhibited IGF-1R phosphorylation. *In vitro* studies suggested that citrate treatment inhibited AKT phosphorylation, activated PTEN and increased expression of p-eIF2a. We also found that p-eIF2a was decreased when PTEN was depleted. These data suggest that citrate acts on the IGF-1R-AKT-PTEN-eIF2a pathway. Additionally, metabolic profiling suggested that both glycolysis and the tricarboxylic acid cycle were suppressed in a similar manner *in vitro* in tumor cells and *in vivo* but only in tumor tissue. We reproduced many of these observations in an inducible Her2/Neu-driven breast cancer model and in syngeneic pancreatic tumor (Pan02) xenografts. Our data suggests that citrate can inhibit tumor growth in diverse tumor types and via multiple mechanisms. Dietary supplementation with citrate may be beneficial as a cancer therapy.

## Introduction

Citrate is an intermediate in the TCA cycle and an essential donor for protein acetylation. Several lines of evidence suggest that citrate may play a role in cancer biology and reduced concentration of citrate in cancer cells may be related to tumor aggressiveness^1^. Citrate exhibits negative feedback on glycolysis^2^ and on the enzyme pyruvate dehydrogenase^3–5^. Our previously published data demonstrated that depletion of ATP-citrate-lyase (ACL) repressed A549 lung cancer cell proliferation *in vitro* and growth *in vivo*, and that this was accompanied by citrate accumulation, suggesting a potential antitumor function for citrate^6^. Similar results have been observed in prostate cancer^7^. *In vitro*, several studies^8–12^ found that citrate reduced proliferation in multiple tumor cell lines. Loss of citrate synthase led to dramatically upregulated glycolysis, decreased citrate production and accelerated tumor growth and metastases^13^. Oral administration of citrate in two patients has been reported to have an antitumor effect, possibly through suppression of glycolysis^14, 15^.

When we began these studies, there was no data on the effects of citrate on tumor growth in animal models. Very recently, a report has been published on the effects of citrate in a gastric cancer model^16^. Here, we report on the therapeutic effects of citrate using diverse xenograft and genetically modified mouse (GEM) models and explore mechanisms of citrate action.

## Results

### Citrate Inhibits Tumor Cell Proliferation In Multiple Tumor Lines

We first tested whether we could reproduce the findings that citrate could inhibit tumor cell proliferation^11, 12^. As shown in Figure S1, citrate significantly inhibited proliferation (Figure S1A–C) and induced cell death (Figure S1D) in lung cancer A549, breast cancer MCF-7 and pancreatic cancer BxPC3 cell lines. Moreover, citrate also induced cell death in two melanoma cell lines, B16F10 and WM983B (Figure S2). Cell lines had different sensitivity to citrate treatment. For example, 5 mM citrate treatment significantly inhibited proliferation of MCF-7 and BxPC3 cell lines. However, for A549 cells, growth inhibition started at 10 mM.

We also tested the effect of citrate on tumor stem cells. Human mammary epithelial cells (HMLEs) were used along with HMLE cells overexpressing Ras (HMLER) or the Snail (Snail) transcription factor. The latter two contain populations of cells that express the cancer stem cell marker phenotype CD44^high^/CD24^low^. As compared to control cells (HMLE cells), cancer stem cells (Snail/Ras-over-expressing) were more sensitive to citrate-induced cell death (Figure S1E). Of note, HMLER cells were intermediate in sensitivity, as might be expected since only some of these cells are CD44^high^/CD24^low^. *In vitro*, citrate treatment appeared to show no selectivity toward cancer cells since it also induced death in non-malignant lung cells at concentrations similar to those that killed tumor cells (Figure S2B).

### Citrate Suppresses Tumor Growth In Xenograft Models

**Figure 1:**
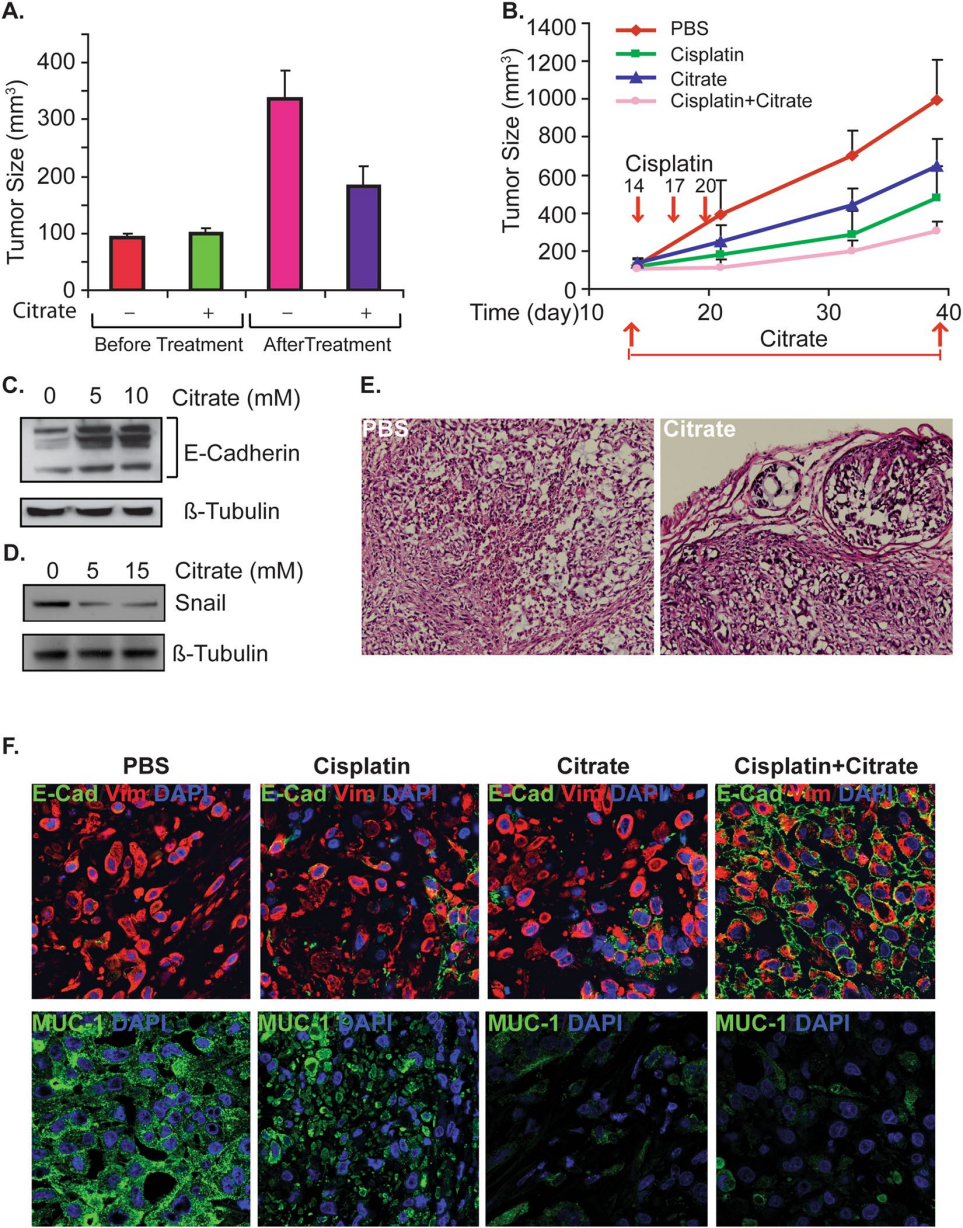
Citrate suppresses tumor growth *in vivo*. The effects of citrate on tumor growth were examined in xenograft and genetic tumor models. (**A**) 5 × 10^6^ A549 cells were injected into nude mice. When the tumor volume reached about 100 mm^3^, mice were given 4 g/kg citrate twice a day for 4 weeks, and tumor-bearing mice were sacrificed after 6 weeks. (**B**) A549 xenograft tumors were treated with citrate (twice daily from day 14 to 40), cisplatin or citrate/cisplatin combination (3 doses of cisplatin at 4 mg/kg on days 14, 17 and 20 were given by i.p.). Tumor volume was calculated using the formula: V = 0.4 AB^2^ (**A**: long diameter; **B**: short diameter). (**C**) A549 cells were treated with citrate for 72 h and cell lysate was analyzed by western blot with E-Cadherin and β-tubulin antibodies. The full-length blots are presented in Supplemental Figure S11. (**D**) A549 cells were treated with citrate for 72 h and cell lysate was analyzed by western blot with Snail and β-tubulin antibodies. The full-length blots are presented in Supplemental Figure S12. (**E**) Citrate treatment induces A549 xenograft tumor differentiation (tumor sections from experiment in panel A). F. 5 × 10^6^ A549 cells were injected into nude mice. Tumor-bearing mice were sacrificed after 6 weeks of treatment and the tumors were dissected and fixed with 4% PBS-balanced PFA and samples were embedded with OCT. E-cadherin and Muc-1 expression was assessed following standard immunofluorescence protocol. DAPI counter staining shows cell nuclei.

To study the effects of citrate on tumor growth *in vivo*, we injected A549 cells subcutaneously into nude mice and examined tumor progression. Mice bearing A549 tumors were randomly divided into treatment and control groups. As shown in Fig. 1A, baseline tumor volumes in the two groups were comparable. Citrate treatment significantly suppressed tumor growth at four weeks compared to the control group.

Cisplatin is commonly used for non-small cell lung cancer (NSCLC) treatment. Citrate treatment has been shown to increase the sensitivity of A549 cells to cisplatin treatment *in vitro* ^8, 11^. To test whether this observation is valid *in vivo*, we examined the treatment effect of cisplatin on A549 tumor growth with or without citrate treatment. As shown in Fig. 1B, both citrate and cisplatin inhibited A549 xenograft tumor growth individually. Citrate treatment along with cisplatin slowed tumor growth even more.

To begin to probe the underlying biological mechanisms, we asked whether citrate treatment led to cell differentiation in A549 cells. Indeed, this appeared to be the case as evidenced by increased E-cadherin expression (Fig. 1C) and reduced snail expression (Fig. 1D) *in vitro*. In A549 xenografts, we observed citrate-induced tumor differentiation with glandular structure formation compared to the diffuse tumor growth pattern in controls (Fig. 1E). Furthermore, while citrate and cisplatin increased E-cadherin expression individually, their combination showed a markedly enhanced effect (Fig. 1F, top panel).

MUC-1 overexpression is associated with poor prognosis in NSCLC and confers resistance to anticancer agents^17, 18^. In A549 xenografts, MUC-1 expression in tumor cells was inhibited by citrate and cisplatin treatment individually, and the combination of citrate and cisplatin further reduced MUC-1 expression (Fig. 1E, bottom panel).

### Citrate Suppresses Tumor Growth In A Lung Cancer GEM Model

**Figure 2:**
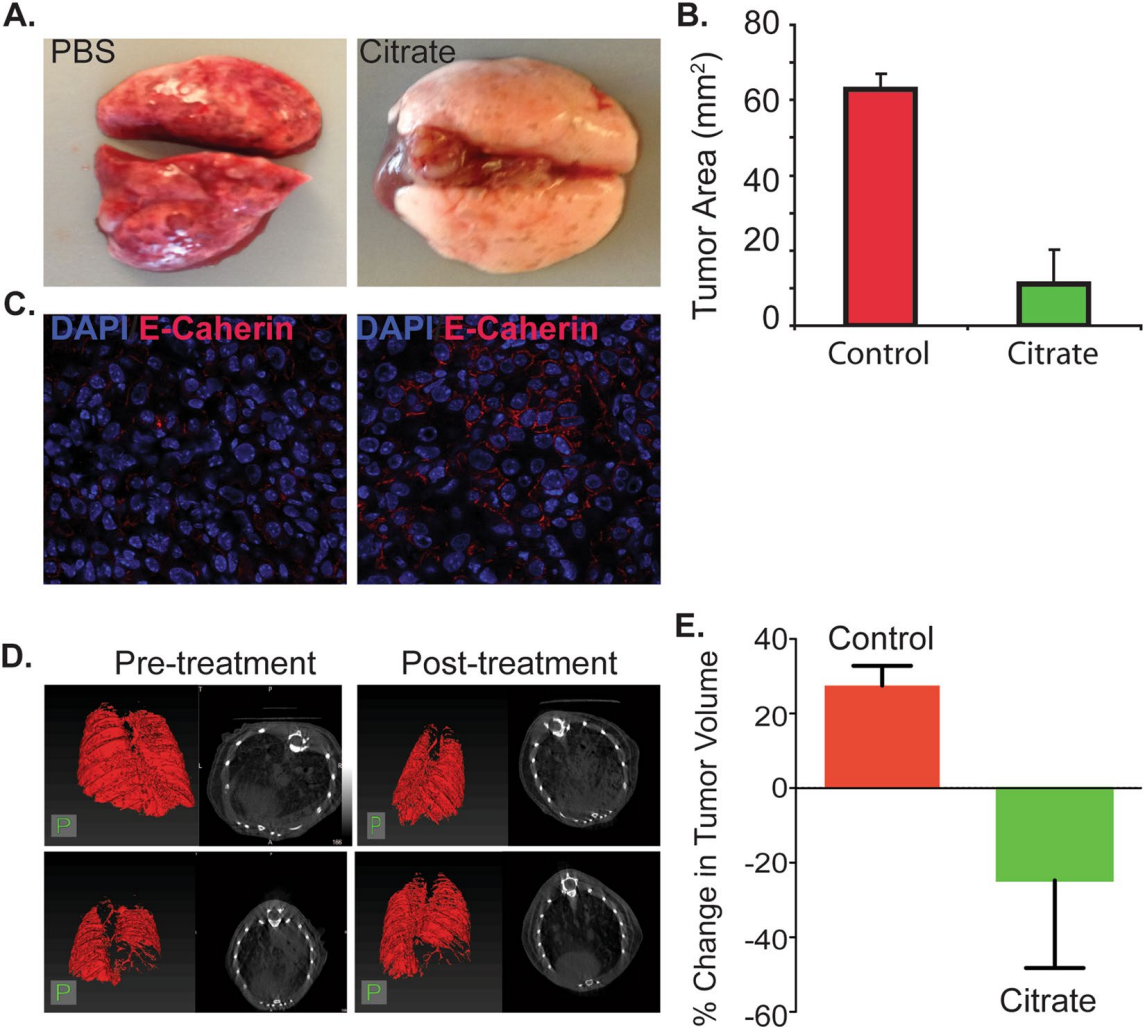
Citrate inhibits Ras driven mouse lung tumor growth in a genetic model. Ras G12D mice with lung tumor were treated with 4 g/kg citrate twice a day for 7 weeks. (**A** and **B**) Citrate treatment affects Ras G12D mouse lung morphology (**A**) and represses tumor growth (**B**). (**C**) citrate induces Ras-driven lung tumor differentiation as indicated by E-cadherin expression. Samples were collected and stained with anti-E-cadherin antibody. (**D**): CT scans on a representative Ras-driven lung tumor before and after citrate treatment. (**E**) Quantitative analysis of lung tumor growth, plotted as percentage change in tumor volume (Control: n = 3; Citrate: n = 4).

Although xenograft tumor models are widely used for cancer studies, the nude mice used in these studies are immunodeficient. Therefore, we used a GEM model of Ras-driven lung tumorigenesis to study the effects of citrate on tumor growth. Mice with Ras-driven tumors were treated with a total of 4g/kg/day of citrate (administered twice a day by gavage) for 7 weeks. As shown in Fig. 2A and B, citrate treatment resulted in a significant reduction in area occupied by tumor nodules (P < 0.001). Consistent with our findings in cell lines and xenografts, citrate induced tumor differentiation in the GEM model as indicated by increased E-cadherin expression (Fig. 2C). Surprisingly, tumor growth suppression in the Ras-driven lung tumor model was more significant than that in the A549 xenograft model, also known to be driven by mutated Ras. Indeed, computerized tomography (CT) scans performed before and after citrate treatment for 7 weeks showed significant tumor shrinkage (Fig. 2D and E). Importantly, at the doses used, there was no evidence of citrate induced tissue toxicity in major organs in the Ras model by histology or function (Figure S3A and Tables S1 and S2). Citrate levels measured in plasma of these chronically citrate treated animals are shown in Figure S3B and are approximately 3 mM, roughly 8 times those noted in non-citrate treated animals. Also, acute treatment of animals with citrate did not show any evidence of toxicity to the liver or kidneys (Tables S3 and S4).

To test whether citrate-mediated tumor suppression extends to other tumor types, we examined the effect of citrate on the growth of breast tumors in a Her2/Neu-driven GEM model. Citrate treatment significantly reduced breast tumor growth (Figures S4A and B), increased E-cadherin expression (Figure S4C), and appeared to induce tumor differentiation as indicated by tubule formation (Figure S4D). Additionally, citrate activated caspase-8 *in vivo* (Figure S4E), consistent with similar *in vitro* data^12^. Besides the growth inhibitory effect of citrate on lung and breast tumors *in vivo*, we also found that citrate administration in drinking water markedly suppressed pancreatic tumor growth (Pan02 cells grown subcutaneously) (Figure S4F). No evidence of toxicity in the liver or lung was noted in the Her2/Neu model with citrate treatment (Figure S5).

### Citrate Promotes Tumor-Infiltrating Leukocytes

**Figure 3:**
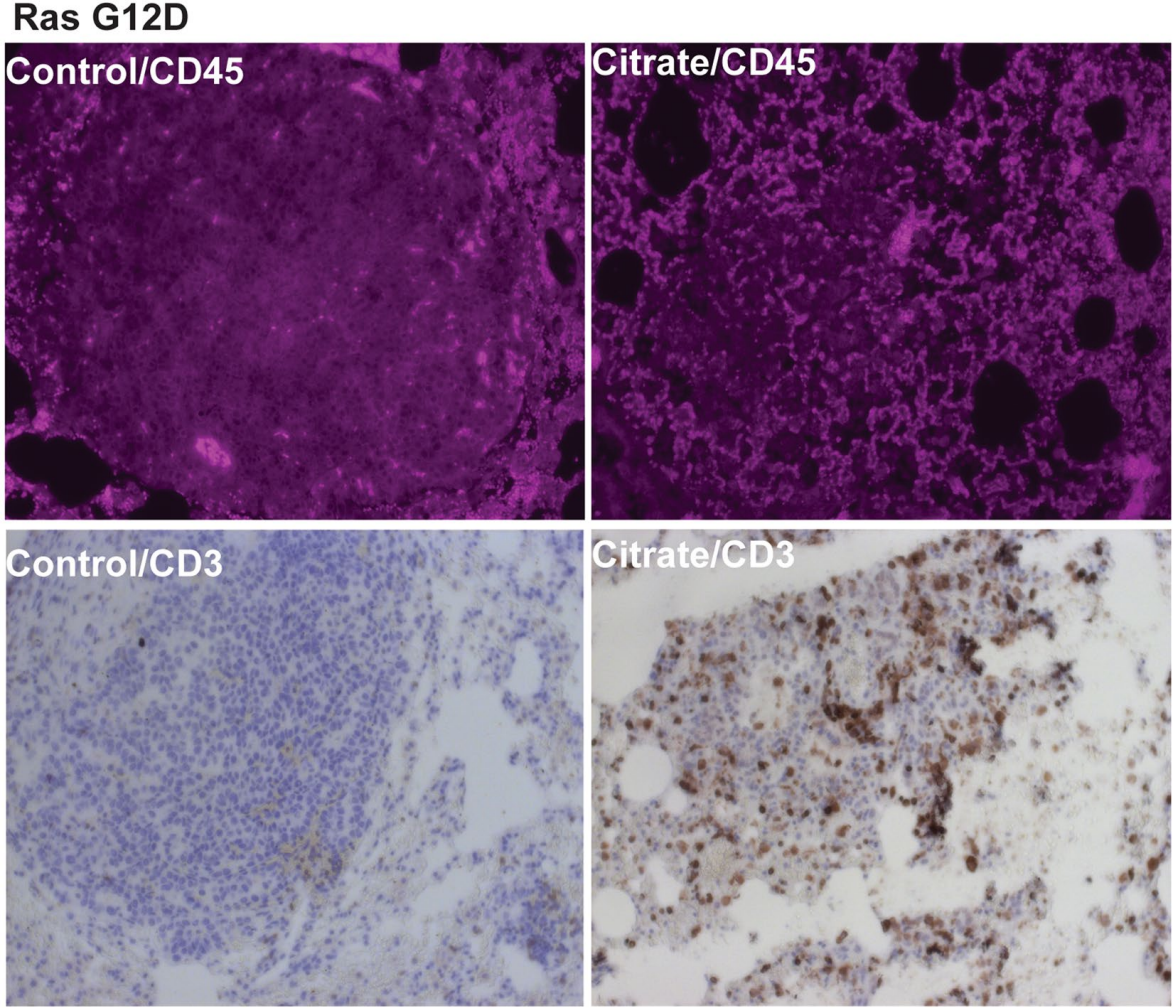
Citrate treatment promotes T-cells infiltration to lung and breast tumor. Ras driven mice with lung tumor were treated with 4 g/kg citrate twice daily for 4 weeks and samples were collected and stained with anti-CD45 and CD3 antibodies.

We noted that tumors in our two genetic models, as compared to xenografts, were more sensitive to citrate treatment. Genetic tumor models have intact immune systems. Citrate metabolism has been reported to play a critical role in lipopolysaccharide (LPS) signaling^19, 20^. Therefore, we asked whether citrate might enhance antitumor immune response. To test this hypothesis, we examined tumor-infiltrating lymphocytes in citrate treated tumor tissue. As shown in Fig. 3 (top panel), in the Ras driven lung tumor model, citrate treated tumors had abundant leukocyte infiltration, as demonstrated by labeling with anti-CD45 antibody. These cells were predominantly T lymphocytes as they were CD3 positive (Fig. 3, bottom panel). Similar results were observed in the Her2/Neu driven breast tumors (Figure S6).

### Citrate Enhances Pro-Inflammatory Cytokine Secretion

**Figure 4:**
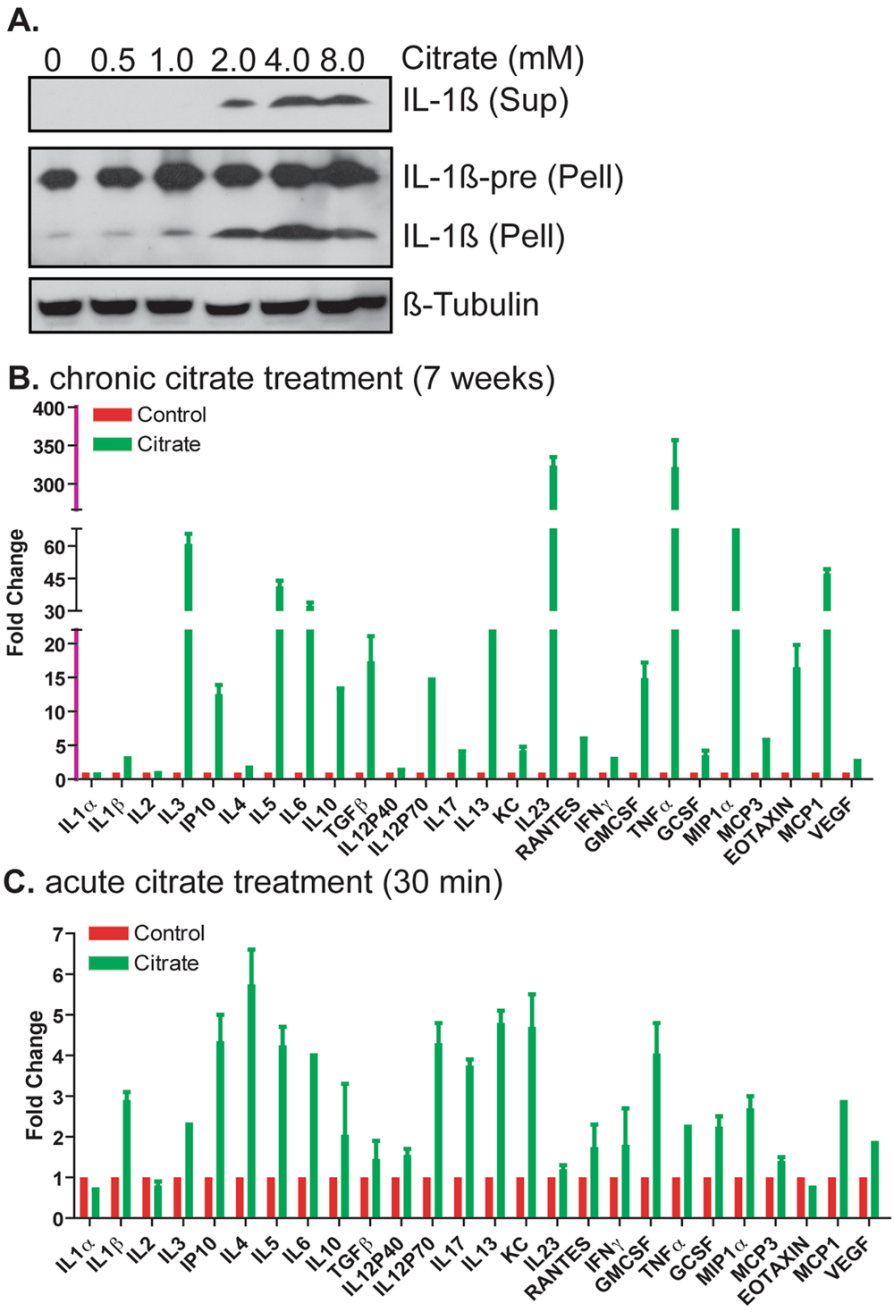
Citrate treatment enhances cytokines secretion. (**A**) Citrate induces IL-1β secretion in PMA-induced macrophages. The full-length blots are presented in Supplemental Figure S13. (**B**) Chronic citrate treatment enhances cytokine secretion in plasma from Ras-driven mice. (**C**) Acute citrate treatment affects cytokine secretion in plasma from Ras-driven mice. Sup: supernatant; Pell: pellet

We asked whether citrate treatment induced secretion of cytokine IL-1β by macrophages *in vitro*, since acute IL-1β secretion regulates immune response and suppresses tumor growth^21^, although chronic IL-1β secretion contributes to tumor development^22^. We treated macrophages (derived from THP1 cells through PMA induction) with citrate and analyzed IL-1β secretion. As shown in Fig. 4A, citrate induced IL-1β secretion in a dose-dependent manner. Next, we asked whether citrate promotes cytokine secretion *in vivo*. We treated Ras-driven lung tumor mice for 7 weeks with 4g/kg citrate and analyzed cytokine profiles in the plasma. As shown in Fig. 4B, 7-week citrate treatment induced the secretion of most cytokines examined, from several fold to several hundred fold. We then examined whether citrate induced cytokines acutely in a GEM. We found that many cytokines increased within 30 min of treatment (Fig. 4C), suggesting cytokine pan-activation. The cytokine pattern for the chronic data was somewhat similar to the acute data but more accentuated. Our data suggest that both pro-inflammatory cytokines (e.g. interleukin-1, tumor necrosis factor-alpha, etc) and anti-inflammatory cytokines (e.g. interleukin-10 and interleukin 1 receptor antagonist) are activated in citrate treated plasma. There was no evidence of Th1 vs Th2 skewing. These results suggest that citrate can rapidly enhance cytokine secretion and that these effects may persist to potentially modulate immune responses *in vivo*.

### Citrate Inhibits IGF-1R Activation And Its Downstream Pathway

**Figure 5:**
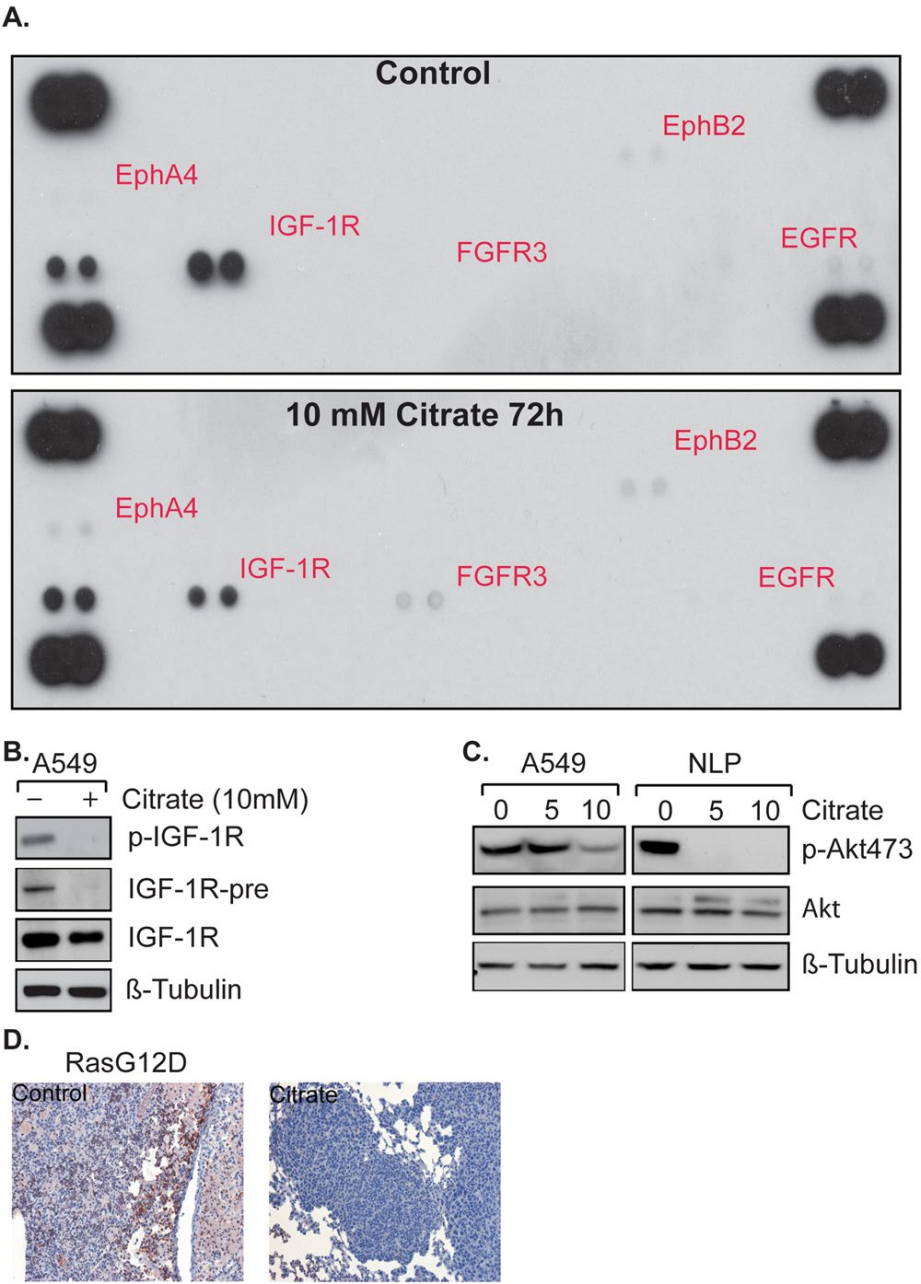
Citrate treatment impacts IGF-1R-AKT pathway. (**A**) A549 cells treated with 10 mM citrate for 72 hours and lysed with lysis buffer provided by the kit and tyrosine kinase changes were screened with the human Phospho-RTK array kit. (**B**) Equal amounts of protein lysate were immunoprecipitated (IP) with anti-IGF-1R antibody and probed with anti-phospho-IGF-1R antibody. The full-length blots are presented in Supplemental Figure S14. (**C**) Citrate treatment inhibits p-AKT in A549 and normal lung epithelial cells. Tumor cells were treated with 10 mM citrate for 72 hours and lysed with RIPA buffer and equal amount of protein were loaded and analyzed by Western Blot with anti-AKT, p-AKT473 and β-tubulin antibodies. The full-length blots are presented in Supplemental Figure S15. (**D**) Immunofluorescence staining for p-IGF-1R citrated treated Ras-driven mice with lung tumor.

To uncover intracellular signaling pathways impacted by citrate treatment, we screened tyrosine kinases targets in A549 cells. As shown in Fig. 5A, tyrosine kinase screening revealed that IGF-1R phosphorylation was significantly reduced in citrate treated cells. To validate this finding, we immunoprecipitated IGF-1R in citrate treated A549 and blotted with anti-p-IGF-1R antibody. Our results confirmed that citrate inhibited IGF-1R phosphorylation in this cell line (Fig. 5B), though this was accompanied by a decrease in total IGF-1R expression. Also, reduced AKT phosphorylation was noted (Fig. 5C). Citrate effects on IGF-1R and AKT phosphorylation were also observed in MCF-7 and BxPC3 cells (Figure S7A), although in these cells, total IGF-1R was unchanged. Curiously, AKT phosphorylation was also diminished by citrate treatment in normal lung primary (NLP) cells (Fig. 5C). To examine whether citrate treatment inhibits IGF-1R phosphorylation *in vivo*, we stained for p-IGF-1R in sections of citrated treated Ras driven lung tumors. As shown in Fig. 5D, citrate inhibited IGF-1R phosphorylation in tumor cells. Similar data was obtained in the Her2/Neu driven breast tumor model (Figure S7B). Durfort *et al*. reported that knockdown of IGF-1R increased the infiltration of lymphocytes and polymorphonuclear neutrophils, and induced secretion of two pro-inflammatory cytokines, TNF-α and IFN-γ^23^. Therefore, we hypothesized that citrate treatment may suppress tumor growth via inhibition of IGF-1R phosphorylation (and activity), which might increase tumor-infiltrating T lymphocytes and pro-inflammatory cytokines.

**Figure 6:**
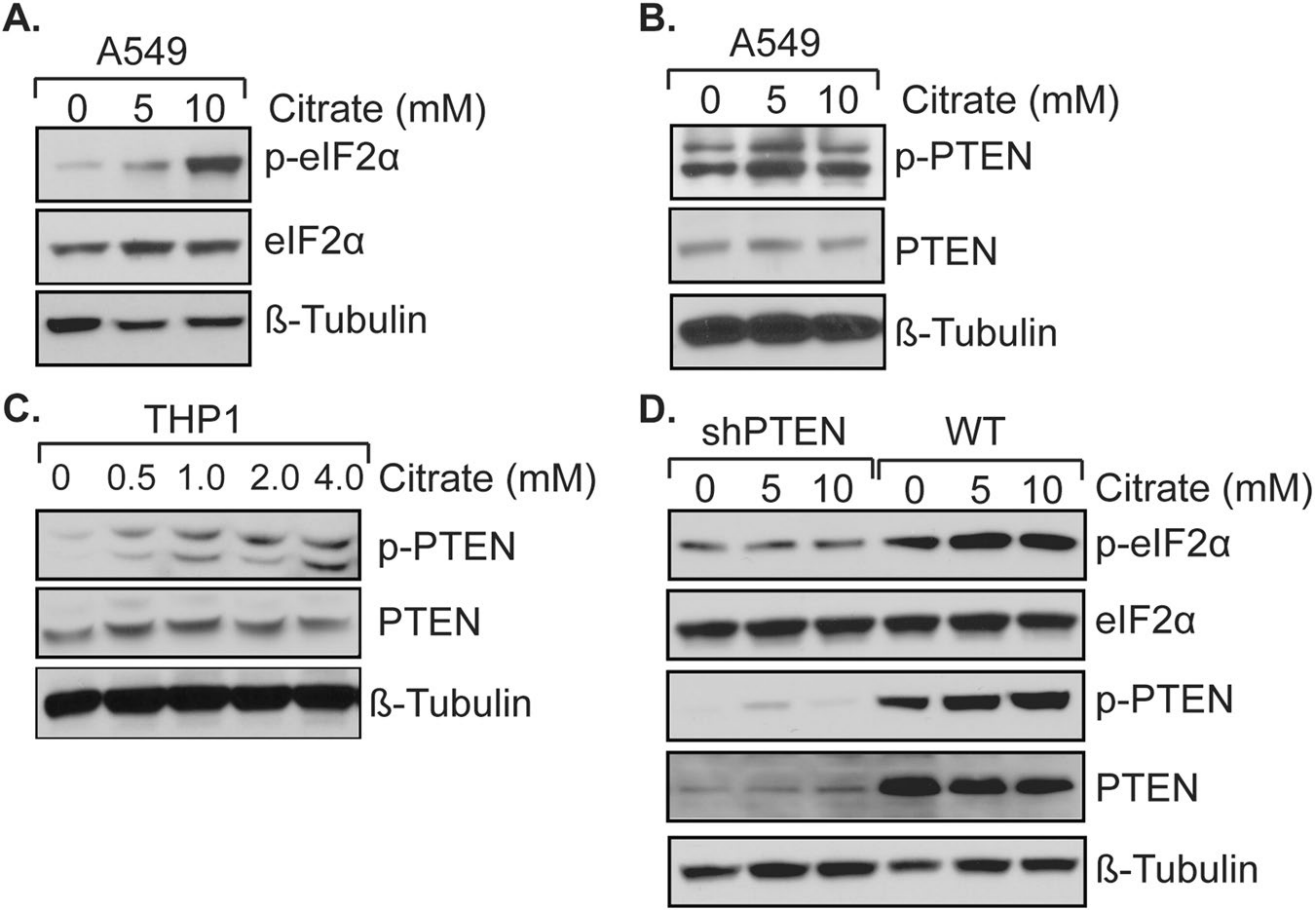
Citrate treatment induces eIF2α expression and activates PTEN pathway. Cells treated with or without citrate for 72 hours and equal amount of protein were subjected to SDS-PAGE. (**A**) Citrate treatment increases e-IF2α activity. The full-length blots are presented in Supplemental Figure S16. (**B**) Citrate treatment increases the active form of PTEN activity in A549 cells. The full-length blots are presented in Supplemental Figure S17. (**C**) Citrate treatment increases the active form of PTEN in a macrophage (THP1) cells. The full-length blots are presented in Supplemental Figure S18. (**D**) Knockdown of PTEN inhibits e-IF2α expression in MCF-10A cells. The full-length blots are presented in Supplemental Figure S19.

To begin to address this question, we examined downstream effectors of the IGF-1R pathway. It is known that IGF-1R activates AKT, which in turn can inhibit PTEN through inactivating the PTEN activator FoX3a via phosphorylation, resulting in inhibition of the PTEN downstream effector eIF2α, an ER stress marker, which is involved in modulating immune responses^24^. Since citrate treatment inhibits IGF-1R and AKT phosphorylation, it may lead to accumulation of non-phosphorylated FoX3a, thus activating PTEN and eIF2α. Thus, we examined eIF2α levels in response to citrate treatment. As shown in Fig. 6A, eIF2α activity was increased in A549 cells after citrate treatment. Similarly, PTEN was activated (as judged by expression of p-PTEN) when A549 cells were treated with citrate (Fig. 6B). The same result was seen in THP1 cells (Fig. 6C). Next, we established cells in which PTEN was stably knocked down using the MCF-10A cell line, and assessed the effect of PTEN on eIF2α expression. As shown in Fig. 6D, in cells in which PTEN was constitutively knocked down by shRNA, depletion of PTEN almost completely blocked eIF2α activation. Taken together, the above data suggest that citrate appears to impact the IGF-1R-AKT-PTEN-eIF2α signal transduction pathway.

### Citrate Represses Glycolysis And TCA Cycle *In Vitro* And *In Vivo*

Increased AKT pathway signaling has been shown to correlate with increased rates of glucose metabolism observed in cancer cells compared to normal cells^25–27^. Conversely, inhibition of glycolysis suppresses tumor growth and can contribute to CD8^+^ T cell maturation^28^. Therefore, we hypothesized that citrate-mediated AKT inhibition might down regulate glycolysis. To probe whether citrate treatment affects glycolysis (as well as the TCA cycle), we examined oxygen consumption and ROS production *in vitro*. Figure S8A shows that citrate treatment of A549 cells dramatically reduced oxygen consumption. Consistent with this data, we observed a decrease in ROS in A549 (Figure S8B) and more dramatically in WM983B melanoma cells (Figure S9). We also measured extracellular acidification rate (ECAR) and found it to be unchanged (data not shown).

**Figure 7:**
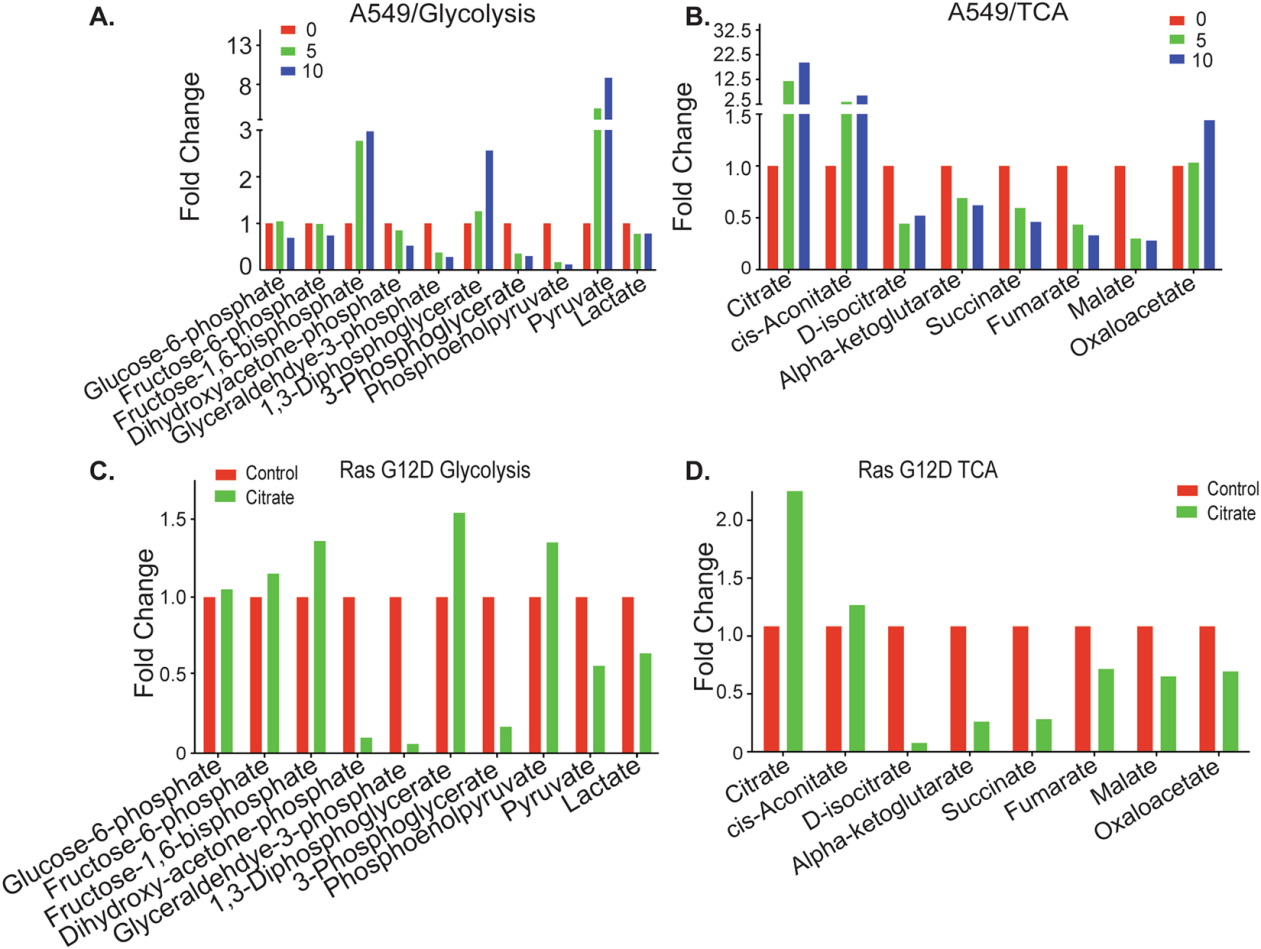
Citrate treatment affects glycolysis and TCA *in vitro* and *in vivo*. (**A**) Citrate treatment affects glycolysis in A549 cells. (**B**) Citrate treatment affects the TCA cycle in A549 cells. (**C**) Citrate treatment affects glycolysis in Ras G12D mouse lung. (**D**) Citrate treatment affects the TCA cycle in Ras G12D mouse lung.

To further explore the effect of citrate treatment on intermediary metabolism, we assessed metabolites both in cell culture and *in vivo*. As shown in Fig. 7A, all glycolytic intermediates except for fructose-1,6-bisphosphate, 1,3-diphosphateglycerate and pyruvate decreased in a dose responsive manner. These data suggest that two glycolytic enzymes may be inhibited by citrate: aldolase which catalyzes conversion of fructose-1,6-bisphosphate to glyceraldehyde 3-phosphate (GLAP) and dihydroxyacetone phosphate, and phosphoglycerate kinase (PGK), which catalyzes conversion of 1,3 diphosphoglycerate to 3-phosphoglycerate. Also, we verified by ^13^C-glucose labeling experiments that the increase in pyruvate is not originating from glycolysis (data not shown) and this increase was not seen *in vivo* (see below). Moreover, we analyzed changes in TCA cycle metabolites in A549 cells treated with citrate. Figure 7B indicates that many TCA cycle metabolites were dramatically reduced, including D-isocitrate, α-ketoglutarate, succinate, fumarate and malate. These changes could be due to inhibition of aconitase, which converts cis-aconitate to D-isocitrate. Collectively, our metabolite profiling indicates that glycolysis and the TCA cycle were inhibited by citrate treatment *in vitro* in A549 cells and is consistent with less oxygen consumption under this condition.

To examine whether these citrate-induced changes also occur *in vivo*, we analyzed metabolic profiles in animals with Ras-driven lung cancers. As shown in Fig. 7C,D, glycolysis and the TCA cycle in the lung tumors appeared to be inhibited, similar to the data *in vitro* in A549 cells, in which Ras is known to be mutated. In particular, changes in glycolytic metabolites mirrored those seen in A549 cells, suggesting blocks at aldolase and PGK. Citrate treatment also appeared to dramatically suppress the TCA cycle. As shown in Fig. 7D, TCA cycle metabolites downstream of cis-aconitate were significantly reduced, similar to the A549 data, suggesting aconitase inhibition. Interestingly, in Ras-driven lung tumors, citrate appeared to affect glycolysis and TCA in the liver only minimally (data not shown). Of note, in the Ras-driven lung tumor model, Ras is over-expressed only in lung tissue and this may explain why citrate treatment leads to different metabolite results in the lung and liver. Taken together, our data suggest that citrate may inhibit tumor growth via inhibiting glycolysis and the TCA cycle and that this effect appears to be selective to tumor tissue.

In Her2/Neu driven breast tumors (Figure S10A), the glycolysis data suggests inhibition of PFK1 (rather than aldolase as noted for the lung tumors) and PGK (similar to lung data) and little impact on the TCA cycle (Figure S10B), also in contrast to the lung tumor data. It seems therefore that citrate affects metabolism somewhat differently in Ras and Her2/Neu driven tumors.

## Discussion

We report several novel findings: (1) citrate administration inhibits the growth of several tumor types (breast, pancreas, lung) both in transplant and genetically engineered models, (2) citrate treatment regresses tumors in a Ras-driven lung cancer model, (3) citrate induces differentiation *in vivo*, (4) citrate alters cytokines in blood and causes T-cell infiltration into tumor tissue, (5) citrate impacts IGF-1R phosphorylation and downstream events including phosphorylation of AKT and eIF2α, (6) citrate suppresses glycolysis and the TCA cycle, acting at specific points in these pathways, with effects *in vitro* and *in vivo* that roughly parallel each other that may be tumor type dependent, and (7) at the doses used, citrate appears to be non-toxic. Collectively our data point to effects of citrate on immune response, tumor metabolism, and signal transduction pathways.

A major motivation for these studies stemmed from our previous work indicating that depletion of ACL leads to citrate accumulation and A549 cell growth inhibition and differentiation. We therefore hypothesized that administration of citrate *in vitro* might mimic the ACL knockdown phenotype^6^. Moreover, several studies had suggested that citrate inhibits tumor cell proliferation and induces apoptosis *in vitro* in multiple cell types. Also, Lin, CC *et al*. found that loss of citrate synthase resulted in markedly unregulated glycolysis, decreased citrate production and accelerated tumor malignancy^13^. Interestingly, citrate concentration in human seminal fluid from prostate cancer patient is 2.7 folds lower than normal patients^29^. Giskeodegard, G.F. *et al*.^30^ studied the metabolic profiling of prostate cancer and found that both the concentrations of spermine and citrate decreased in high grade prostate tumor samples as compared with normal tissues. Along with two patient reports cited earlier, these studies suggested that we should explore the efficacy of citrate treatment in various tumor models and probe possible mechanisms of action.

Citrate’s effect in several tumor models may be explained by its broad actions on signaling pathways (in particular those involving IGF-1R and its downstream events), intermediary metabolism (both glycolysis and TCA cycles), and immune response (T-cell infiltration and cytokine changes). These multiple mechanisms of action may explain the ability of citrate treatment to regress Ras-transformed tumors. Regression is not often seen with interventions that inhibit a single intermediary metabolic enzyme (one exception is inhibition of LDH-A^31^; another is IDH-1 in leukemic cells^32^).

The ability of citrate treatment to promote differentiation and reverse EMT *in vivo* especially in conjunction with chemotherapy (as assessed by E-cadherin, vimentin and MUC-1 expression) is also noteworthy. The effects on MUC-1 are especially impressive since a large body of literature suggests that MUC-1 expression is indicative of an aggressive cancer phenotype^33, 34^.

Our data suggest that part of citrate’s efficacy is via the immune system. Citrate can promote a striking increase across a wide range of cytokines, almost reminiscent of a cytokine storm. Disappointingly, there was no clear-cut polarization of the response e.g. a skewing to the Th1 direction. Drugs that might do this might synergize with citrate therapy. Why does citrate display the immune-enhancing function? IGF/IGF-1R plays important and diverse roles in tissue development and function. This pathway is also involved in immune function regulation^35^. Knockdown IGF-1R delays tumor growth and induces proinflammatory cytokines in a mouse breast cancer model^23^. The number of infiltrating T-cells significantly increased in citrate treated tumor samples, suggesting activation of antitumor adaptive immune response. Current experiments are defining the cell types involved in the immune response, the impact of ablating specific T cell populations and the possibility of combining citrate therapy with other immune interventions such as checkpoint inhibitors. It will also be important to define the effects of citrate on T cell metabolism (glycolysis and TCA cycle), since there could be both beneficial and detrimental effects e.g. Sukumar, M. *et al*.^28^ found that augmenting glycolytic flux drives CD8^+^ T cells toward a terminal differentiated state, while its inhibition preserves the formation of long-lived memory CD8^+^ T cells.

The IGF-1R pathway appears to plays an important role in tumorigenesis, metastasis and resistance to existing forms of anti-cancer therapy^36, 37^. Especially in NSCLC and triple-negative breast cancer cell lines, high IGF-1R activity correlates with sensitivity to anti-IGF-1R therapy^38, 39^. Many preclinical studies targeting the IGF-1R have been shown promising anti-neoplastic activity^40^ and early phase I^41^ and phase 2^42^ reports have been encouraging. We found that citrate inhibits IGF-1R activity *in vitro* and *in vivo*. The mechanism by which citrate treatment leads to IGF-1R activity inhibition is uncertain. Our data suggested that citrate treatment inhibits both IGF-1R and AKT activity in multiple cells lines. This means that citrate may affect the IGF-1R/AKT signal transduction pathway. Decreased AKT activity could inhibit FoxO3a phosphorylation and lead to FoxO3a accumulation in the nucleus, which can upregulate PTEN^43^. Our data supports this hypothesis, since we found that citrate treatment increased PTEN activity in A549 cells. eIF2α phosphorylation is important to response to various types of environmental stress and is essential for regulation of translation initiation. Phosphoinositide-3-kinase (PI3K) plays an important role in signal transduction in response to a wide range of cellular stimuli involved in cellular process that promote cell proliferation and survival. The eIF2α phosphorylation pathway is downstream of PTEN, and reconstitution of PTEN-null human glioblastoma or prostate cancer cells with either wild-type PTEN or phosphatase-defective mutants of PTEN induced eIF2α phosphorylation^44^. It has been shown that deregulation of the eIF2α checkpoint and consequent permissiveness to virus infection may be a common occurrence in tumorigenic mammalian cell lines^45^. In our system, the phosphorylation of eIF2α dramatically increased when treated with citrate in A549 cells. Knockdown of PTEN in a breast tumor cell line inhibited the phosphorylation of eIF2α. Collectively, our study indicates that citrate treatment activates eIF2α through down regulating the IGF-1R-AKT pathway and up regulating the PTEN- eIF2α pathway.

Citrate is also a key metabolite known to exert a negative feedback on glycolysis via allosteric modulation of PFK1. ATP inhibits the enzyme by decreasing its affinity for fructose-6-phosphate. Citrate enhances the inhibitory effect of ATP^3^. Our findings are that citrate can impact glycolysis in tumor tissue at different points (PFK1, aldolase, PGK) and that this may depend on the driving mutations and tumor type. Also, our findings suggesting inhibition of aconitase (in the TCA cycle) in the Ras model are novel.

Very recently, Wang *et al*.^16^ have reported *in vivo* data using citrate in a gastric cancer model. They showed decreased glycolysis *in vitro* and increased apoptosis *in vitro* and *in vivo*. Interestingly, they used significantly lower doses of citrate (the maximum dose was 30 mg per kilogram per day) and obtained inhibition of approximately 50% in tumor volume. They noted that PFK1 enzyme activity was inhibited by 60 to 70% *in vitro*, as suggested by our data in Her2/Neu mice. Curiously, IGF-1R is highly expressed in some gastric cancers and may explain sensitivity of this tumor type to citrate therapy^46, 47^.

Chronic citrate treatment was non-toxic as evidenced by gross pathology in numerous organs (liver, lung, spleen and kidney) and by plasma electrolytes and liver and kidney function tests. Plasma levels in chronically treated animals were approximately 3 mM and of note, calcium levels were unchanged. Both gavage twice a day and drinking water throughout the day appeared to be effective (total dose 8 g per day), corresponding to approximately 56 g of citrate in a 70 kg person. A phase 1 study using 0.5 g per kilogram of sodium citrate given orally was well-tolerated^48, 49^.

Our study has several limitations. We do not know how the exogenously administered citrate is transported into tumor and normal cells and how it affects intracellular citrate and in what cell compartments. Which of the effects of citrate are the most important in affecting tumor biology? Does citrate impact systemic pH as part of its mechanism of action? What appears to give this therapy selectivity for tumor cells? Despite these limitations, our data suggests that clinical studies using citrate to treat a wide variety of cancers are warranted as a non-toxic and affordable treatment.

## Materials and Methods

### Materials

Dichlorodihydrofluorescein diacetate (CM-H2DCF-DA) was purchased from Invitrogen/Molecular Probes (Carlsbad, CA). Fetal bovine serum (FBS) was obtained from GIBCO. The anti-E-cadherin monoclonal antibody was from Santa Cruz Biotechnology. The Caspase-8, 3 and 9, anti-IGF-1R, phosphor-IGF-1R, phospho-AKT473, AKT1/2, phosphor-AMPKα (T172) and AMPKα polyclonal antibodies were purchased from Cell Signaling Technology. The anti CD3 and anti-CD45 antibodies were from Abcam. Secondary antibodies for enhanced chemiluminescence (ECL) detection were from Amersham Biosciences. Citrate assay kit was obtained from Abcam. Human Phospho-Receptor Tyrosine Kinase Array Kit was purchased from R&D systems. All other reagents were of standard analytical grade.

### Cell Culture

The human lung cancer cell line A549, MCF-7, BxPC3, B16F10 and normal human lung epithelial cells were obtained from American Type Culture Collection. WM983B melanoma cells were purchased from the Wistar Institute. A549 cells were grown in Hams/F12 Medium (Cellgro, VA). MCF-7 cells were maintained in DMEM medium. BxPC3 and B16F10 cells were grown in RPMI 1640 medium. All of the above media were supplemented with 10% (v/v) fetal calf serum, 100 units penicillin and 100 μg/ml streptomycin, and grown at 37 °C and 5% CO_2_. WM983B melanoma cells were grown in MCDB153 medium and supplemented 20% horse serum and 2% FBS. The normal lung epithelial cells were grown in Ham’s F-12 medium plus growth factors. HMLE, HMLER and Snail cells (HMLE cells with constitutive expression of mutated Ras and Snail respectively) were grown in mammary epithelial growth (MEGM) medium (Biowhittacker, Inc., Maryland, MD, USA), a serum-free medium composed of modified MCDB 170 basal medium with supplements) as described before^50^.

### Western Blotting

Cells with and without citrate addition were lysed with RIPA buffer (50 mM Tris-HCl, pH 7.4, 150 mM NaCl, 1% NP-40, 0.1% SDS and 0.5% sodium deoxycholate), and equal protein amounts were resolved by 4–12% Bis-Tris gels (Invitrogen), as previously described^51^. Briefly, the proteins were transferred to a PVDF membrane, and membranes were blocked with BLOTTO (5% nonfat dry milk and 0.1% Tween 20 in PBS), and incubated with antisera. Membranes were washed in PBS plus 0.1% Tween 20, probed with anti-rabbit or anti-mouse HRP-conjugated secondary antibody (both at 1:10,000 dilution), and proteins were detected using the ECL Plus chemiluminescence detection reagent (Amersham Biosciences).

### Human Phospho-Receptor Tyrosine Kinase Array

The Human Phospho-receptor tyrosine kinase array kit was used to screen changes of phospho-receptor tyrosine kinase when treated with citrate in A549 cells. Briefly, A549 cells were treated with 10 mM citrate for 72 hours and lysed with lysis buffer from the kit and incubated with the array provided by the kit following their instructions.

### Immunoprecipitate IGF-1R

To validate whether citrate affects IGF-1R, A549 cells were treated with or without citrate for 72 h, and lysed with lysis buffer (20 mM Tris-HCl, pH 7.5, 137 mM NaCl, 0.5% Triton X-100, and 10% Glycerol). Equal amount of protein was incubated with anti-IGF-1R antibody and protein A/G beads for 2 h at 4 °C, and then washed three times with washing buffer. Samples were resolved by SDS/PAGE and Western blotting and probed with anti-p-IGF-1R antibody

### Citrate Assay

The concentration of mouse plasma citrate was determined by a citrate assay kit following the manual (Abcam). Briefly, plasma from chronic citrate treated mice was deproteinized using 10 kDa molecular weight cut off spin columns and incubated with 50 μl citrate reaction mixture containing enzyme mix, developer and citrate probe for 30 min at room temperature. Citrate content was measured by reading OD 570 nm.

### Cytokines Assay

The effect of citrate on plasma cytokines was analyzed by Luminex (polystyrene bead kits). Ras-driven lung cancer mice were chronically treated with 4 g/kg citrate (twice a day) for 7 weeks. For certain experiments, 30 min before sacrifice, mice were dosed with citrate and plasma was collected. This assay was performed in the Human Immune Monitoring Center at Stanford University. Mouse 26 plex kits were purchased from Affymetrix and used according to the manufacturer’s recommendations with modifications as described below. Briefly, samples were mixed with antibody-linked polystyrene beads on 96-well filter-bottom plates and incubated at room temperature for 2 h followed by overnight incubation at 4 °C. Room temperature incubation steps were performed on an orbital shaker at 500–600 rpm. Plates were vacuum filtered and washed twice with wash buffer, then incubated with biotinylated detection antibody for 2 h at room temperature. Samples were then filtered and washed twice as above and resuspended in streptavidin-PE. After incubation for 40 minutes at room temperature, two additional vacuum washes were performed, and the samples resuspended in Reading Buffer. Each sample was measured in duplicate. Plates were read using a Luminex 200 instrument with a lower bound of 100 beads per sample per cytokine.

### Proliferation Assay

Control and citrate treated cell were plated in 6-well plate at a density of 1 × 10^5^ cells/well and maintained at 37 °C in a 5% CO_2_ incubator. After 24, 72, 120 and 168 hours of initial plating, 0.5 ml cells were diluted into 10 ml of Hanks’ buffer and counted by Coulter counter. All samples were assayed in triplicate to generate proliferation curves as described^52^.

### Annexin-V Apoptosis Assay

Apoptosis was measured by staining with the Nexin reagent using a Nexin kit and counting on the Guava PCA-96 system (Guava Technologies) as per the manufacture’s protocol. Briefly, cells were harvested and re-suspended in 100 µl of 1X Nexin buffer, and then mixed with 100 µl of Annexin-V-PE, and Nexin 7-AAD. The cells were allowed to incubate for 20 minutes at room temperature and analyzed in the Guava flow cytometer.

### Determination of Cellular Reactive Oxygen Species (ROS)

Intracellular ROS production was measured by staining with CM-H2DCFDA. CM-H2DCFDA is a cell-permeant indicator for ROS that is non-fluorescent until removal of the acetate groups by intracellular esterases and oxidation occurs within the cell. The procedure for measuring ROS was carried out as described earlier, with minor modification^53^. Briefly, A549 cells transduced with shRNA lentiviral particles or control vector were selected with puromycin for 2 weeks, and then incubated with 10 µM CM-H_2_DCF-DA for 3 hours, followed by flow cytometry using a FACSCalibur equipped with CellQuest Pro software. Superoxide radicals (O_2_^−^) were measured separately using the MitoSOX reagent according to the manufacturer’s protocol (Invitrogen). In brief, cells with or without ME2 knockdown were incubated with 5 µM MitoSOX^TM^ reagent for 10 minutes at 37 °C, then washed three times and observed under a fluorescence microscope using the Rhodamine filter and Axiovision software for capturing images (Zeiss, Germany).

### Xenograft Model in Nude Mice

Animal experiments were performed under federal guidelines and approved by the Institutional Animal Care and Use Committee (IACUC) of the Beth Israel Deaconess Medical Center (approval number 0342007). A549 xenografts in nude mice were generated by following the description of Verrax J *et al*.^54^. Briefly, approximately 5 × 10^6^ A549 cells suspended in 100 µl of a serum-free culture medium were subcutaneously injected into the right and left flanks of female Athymic nude mice, respectively. Tumor-bearing mice were sacrificed after 6–8 weeks and tumor masses were measured or imaged before excision. Tumor lysates were prepared by homogenization of tumor tissues in RIPA lysis buffer and were resolved by SDS-PAGE and transferred onto PDVF membranes and immunoblotted with various antibodies and normalized by ß-tubulin as a loading control.

### K-RasG12D driven lung tumor

The Ras-driven mouse lung tumor transgenic mice were generated following Fisher, G.H. *et al*.^55^, and housed in pathogen-free conditions and handled in accordance with institutional guidelines. Briefly, bitransgenic mice were produced by crossing the *CCSP-rtTA* activator mice to *Tet-op-K-RasG12D* responder mice. All genotyping was done by PCR as described in the literature. Once homozygous K-RasG12D mice were obtained, the mice were switched to tetracycline water and continued to be housed for 1–2 months, and then treated with 4 g/kg citrate twice a day by gavage. In order to monitor tumor volume change, before and after citrate treatment, the tumors in the lung were scanned using micro CT method as described^31^. Briefly, animals were anesthetized with 2% isoflurane/balance O2. Imaging was performed using the CT component of a NanoPET/CT (Bioscan, Washington, DC) scanner equipped with an 8 W X-ray source running at 45kVp (178mA) and a 48 mm pitch CMOS-CCD X-ray detector. Continuous helical micro-CT scanning was employed with the following parameters: 1.6 s exposure, 483 angles, 2.45 magnification, 37 mm pitch (1 field-of-view), and a 512 × 1798 pixel frame size (0.096 mm pixels). Images were reconstructed as 536 × 536 pixel transverse matrices with varying axial length and slice thickness of 0.1mm (isotropic voxel size 0.078 mm) using filtered-back projection (RamLak filtering). Image analysis was performed using VivoQuant software (inviCRO, Boston, MA) using a Hounsfield Unit windowing technique, normalized for all scans. Volumetric segmentation was performed using a neighborhood threshold^31^.

### Her2/Neu Driven Mouse Breast Tumor

The Her2/Neu-driven mouse breast tumor transgenic mice were generated as described before^56^. Briefly, transgenic mice were housed in pathogen-free conditions and handled in accordance with institutional guidelines with a 12 hr light/dark cycle and access to food and water ad libitum. Induced animals were administered doxycycline (0.1–2 mg/ml) (Sigma) in their drinking water, which was replaced weekly. Animals were inspected for tumors, and existing tumors were measured weekly and treated with 4 g/kg citrate by gavage twice a day. At the indicated times of sacrifice, animals were killed by CO_2_ asphyxiation and tissues were either snap-frozen on dry ice for protein analysis, or fixed in 4% paraformaldehyde for morphological and immunohistochemical analysis.

### Metabolite Profiling

To determine differences in metabolite profiles between citrate-treated and control cells, metabolite extracts were prepared and then analyzed using liquid chromatography tandem mass spectrometry (LC-MS) as described before^57, 58^.

### Tumor Area Measurement And Lung Metastasis Assay

Lungs from mice bearing Ras-driven tumors were removed and washed three times with PBS. Formalin-fixed, paraffin-embedded mouse lung tissue sections were prepared at 5 μm. H&E stained slides were scanned and tumor area were quantified with Image J (NIH software). Microscopic images (40X) of stained tissue sections were collected. Similarly, lung metastatic lesions were counted under a low-power microscope. Statistical significance was analyzed using PRISM software.

### Statistical Analysis

All data are expressed as the mean ± s.d. and were analyzed using the two-tailed Student t-test or one-way ANOVA. P < 0.05 was considered to be significant. *P < 0.05; **P < 0.01; ***P < 0.001. All tests were performed using the PRISM software (GraphPad Software, Inc., La Jolla, CA).

## Electronic supplementary material

Supplementary Information
