# Supplementary material for: Citrate Suppresses Tumor Growth in Multiple Models through Inhibition of Glycolysis, the Tricarboxylic Acid Cycle and the IGF-1R Pathway
Source: Sci Rep. 2017 Jul 3;7:4537. doi: 10.1038/s41598-017-04626-4 (PMC5495754; doi:10.1038/s41598-017-04626-4)
Supplement: Supplementary file 1 — Supplementary Information [file 41598_2017_4626_MOESM1_ESM.pdf]

SUPPLEMENTARY INFORMATION

Citrate Suppresses Tumor Growth in Multiple Models through Inhibition of Glycolysis, the Tricarboxylic Acid Cycle and the IGF-1R Pathway

Jian-Guo Ren[1], Pankaj Seth[1], Huihui Ye[2], Kun Guo[1,3], Jun-ichi Hanai[1], Zaheed Husain[1] and Vikas P. Sukhatme[1]*

[1]Divisions of Interdisciplinary Medicine and Biotechnology, Hematology-Oncology and Nephrology, Department of Medicine and the Cancer Research Institute, [2] Department of Pathology, Beth Israel Deaconess Medical Center (BIDMC) and Harvard Medical School, 330 Brookline Avenue, Boston, MA 02215; [3]Zhongshan Hospital, Fudan University, Shanghai, China, 200032.

* Correspondence: Vikas P. Sukhatme MD ScD

Beth Israel Deaconess Medical Center.

330 Brookline Avenue, Gryzmish 602, Boston, MA 02215

Tel: 617-667-9050

Fax: 617-667-7843

E-mail: vsukhatm@bidmc.harvard.edu

Running title: Citrate suppresses tumor growth

The authors declare no potential conflicts of interest.

SUPPLEMNTAL TABLES AND FIGURE LEGNEDS.

Table S1. The effect of chronic citrate treatment on liver function. Blood from mice treated for several weeks with citrate at 4 g/kg twice a day was assayed for liver function tests.

Table S2. The effect of chronic citrate treatment on kidney function.

Table S3. The effect of acute citrate treatment on liver function. Blood was sampled at the times indicated following one time citrate administration at 4 g/kg by gavage.

Table S4. The effect of acute citrate treatment on kidney function.

Figure S1. Citrate inhibits human tumor growth and induces cell death in vitro. Tumor cell lines were treated with different concentrations of citrate for 72 h and cells were counted and cell death was analyzed by Annexin V kit. A: Citrate inhibits A549 cell growth. B. Citrate inhibits BxPC3 cell proliferation. C. Citrate inhibits MCF-7 cell proliferation. D. Citrate induces A549, BxPC3 and MCF-7 cell death. E. Citrate induces cell death in HMLE, HMLER and Snail cells.

Figure S2. Citrate induces cell death in vitro. Tumor cell lines were treated with different concentrations of citrate for 72 h and cell death was analyzed by flow cytometry. A: Citrate induces WM983B and B16F10 cell death. B. Citrate induces Normal lung primary (NLP) and BEAS2B cell death.

Figure S3. A. The effect of citrate on Ras-driven mouse organ pathology as seen by H and E staining. Liver, kidney, spleen and lung tissue showing no gross abnormalities. B. Plasma citrate concentration in chronically citrate treated animals and controls.

Figure S4. Citrate inhibits Her2/Neu driven mouse breast tumor growth. A: Image of excised tumors in citrate treated and control mice. B: tumor weight in citrate treated and control mice. C: Citrate induces Her2/Neu-driven tumor differentiation as indicated by E-cadherin expression. Her2/Neu mice with lung tumor were treated with 4g/kg citrate twice daily for 7 weeks and samples were collected and stained with anti-E-cadherin antibody. D: Citrate treatment induces Her2/Neu driven mouse breast tumor differentiation. E: Citrate induces caspase-8 activation in vivo. F: Citrate treatment affects Pan02 mouse pancreatic tumor growth. Here citrate was dissolved in PBS containing 5% sucrose and administered in drinking water. Citrate amount dissolved corresponded to 4g/kg by gavage daily assuming 2 ml daily input of drinking water.

Figure S5. The effect of citrate on Her2/Neu-driven mouse lung and liver organ pathology. No gross changes were noted in citrate treated animals in lung or liver tissue.

Figure S6. Citrate treatment promotes T-cells infiltrating to breast tumor. Her2/Neu driven mice with breast tumor were treated with 4g/kg citrate twice daily for 4 weeks and samples were collected and stained with anti-CD3 antibody.

Figure S7. Citrate treatment impacts the IGF-1R-AKT pathway. A. Citrate inhibits p-IGF-1R and AKT activity in MCF-7 and BxPC3 cells as detected by Western blotting. B. Immunofluorescence staining for p-IGF-1R citrate treated Her2/Neu-driven mice. C. Quantitative analysis of p-IGF-1R positive cells in Her2/Neu tumor tissues. The images were captured under 20x, and positive p-IGF-1R cells were counted and expressed as the average per field. At least three sections in each sample were analyzed. ***: $p<0.001$ as compared with control.

Figure S8. Citrate inhibits oxygen consumption and ROS production. A. A549 were treated with 10 mM citrate for 24 hours and oxygen consumption was measured by a Seahorse machine. B. Decreased ROS in A549 cells was noted by flow cytometry using CM-H2DCFDA as a probe. Each histogram is representative of three experiments.

Figure S9. Citrate inhibits oxygen consumption and ROS production. Decreased ROS in WM983B cells detected by flow cytometry using CM-H2DCFDA as a probe. Histograms are representative of three experiments.

Figure S10. Citrate treatment affects glycolysis and TCA in vivo in the Her2/Neu model. Metabolite profile for glycolysis (A) and the TCA cycle (B) in tumor tissue.

Figure S11. Full length blots of Figure 1C.

Figure S12. Full length blots of Figure 1D.

Figure S13. Full length blots of Figure 4A.

Figure S14. Full length blots of Figure 5B.

Figure S15. Full length blots of Figure 5C.

Figure S16. Full length blots of Figure 6A.

Figure S17. Full length blots of Figure 6B.

Figure S18. Full length blots of Figure 6C.

Figure S19. Full length blots of Figure 6D.

Table S1: Chronic treatment liver function assay

Treament	Control	Citrate	Reportable Ranges
Total Bilirubin	0.96±0.12	1.87±0.63	0-10.0mg/L
Total Protein	4.2±0.1	7.15±2.09	2-11 g/dL
Albumin	3.75±0.12	5.23+0.75	1.0-6.0 g/dL
Aspartate Aminotransferase (AST)	165.34±23.87	207.56±17.21	4-950 U/L
Alanine Aminotransferase (ALT)	21.2±2.51	9.87±0.24	3-950 U/L

Table S2: Chronic kidney function assay

Treatment	Control	Citrate	Reportable Ranges
Creatinine	0.3±0.01	0.2±0.01	0.2-11.2 mg/dL
BUN (Blood Urea Nitrogen)	28±0.04	25±6.34	1-100 mg/dL
Calcium	2±0.1	2.5±0.05	3-14 mg/DL
Carbon Dioxide	17.1±0.91	22±1.02	5-50 mmol/L
Chloride	129.2±3.54	121±2.71	65-140 mmol/L
Glucose	218.1±14.78	350±136.55	20-450 mg/dL
Phosphorous	7.5±0.12	5.43±0.08	0.5 -13.0 mg/dL
Potassium	11±0.01	11±0.01	1-11 mmol/L
Sodium	137.5±2.34	142.56±8.23	95-215 mmol/L

Table S3: Acute liver function assay

Treatment Time (min)	0	30	120	Reportable Ranges
Total Bilirubin	4.45±3.32	3.9±1.13	2.9±0.566	0-10.0mg/L
Total Protein	7.5±1.7	8.4±1.13	8±0.990	2-11 g/dL
Albumin	4.85±1.63	5.25±1.06	4.75±0.354	1.0-6.0 g/dL
Aspartate Aminotransferase (AST	133.5±58.69	156.5±0.71	230±53.74	4-950 U/L
Alanine Aminotransferase (ALT)	18±0.3	21.5±26.16	73.5±26.163	3-950 U/L

Table S4: Acute kidney function assay

Treatment Time (min)	0	30	120	Reportable Ranges
Creatinine	0.2±0.01	0.2±0.01	0.2±0.01	0.2-11.2 mg/dL
BUN (Blood Urea Nitrogen)	26±0.01	27±5.66	15±0.02	1-100 mg/dL
Calcium	3±0.02	3±0.02	3±0.02	3-14 mg/DL
Carbon Dioxide	13.5±0.71	19±1.41	22±0.01	5-50 mmol/L
Chloride	120.5±2.12	124±1.41	130±0.01	65-140 mmol/L
Glucose	233±15.56	332±166.88	135±46.67	20-450 mg/dL
Phosphorous	7.9±0.28	4.95±0.07	6.25±1.061	0.5 -13.0 mg/dL
Potassium	11±0.01	11±0.01	11±0.01	1-11 mmol/L
Sodium	143.5±0.71	149±4.24	153±4.24	95-215 mmol/L

Figure S1

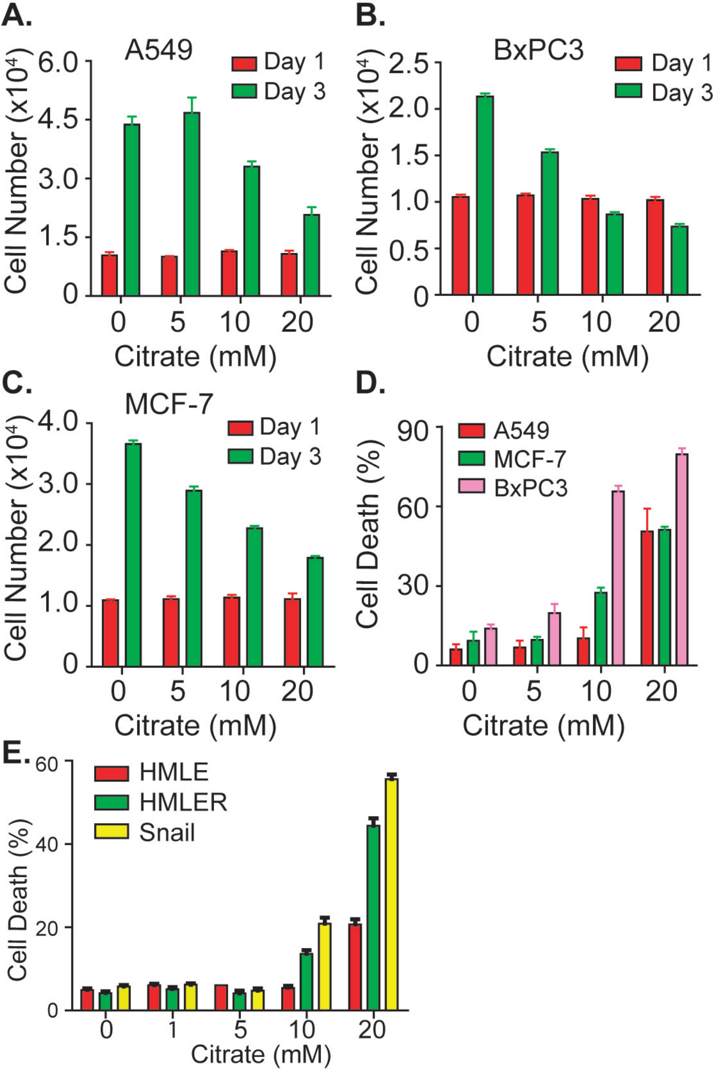
A.
A549
Day 1
Day 3
Cell Number (x10^4)
6.0
4.5
3.0
1.5
0
0
5
10
20
Citrate (mM)
B.
BxPC3
Day 1
Day 3
Cell Number (x10^4)
2.5
2.0
1.5
1.0
0.5
0
0
5
10
20
Citrate (mM)
C.
MCF-7
Day 1
Day 3
Cell Number (x10^4)
4.0
3.0
2.0
1.0
0
0
5
10
20
Citrate (mM)
D.
A549
MCF-7
BxPC3
Cell Death (%)
90
60
30
0
0
5
10
20
Citrate (mM)
E.
HMLE
HMLER
Snail
Cell Death (%)
60
40
20
0
0
1
5
10
20
Citrate (mM)

Figure S2

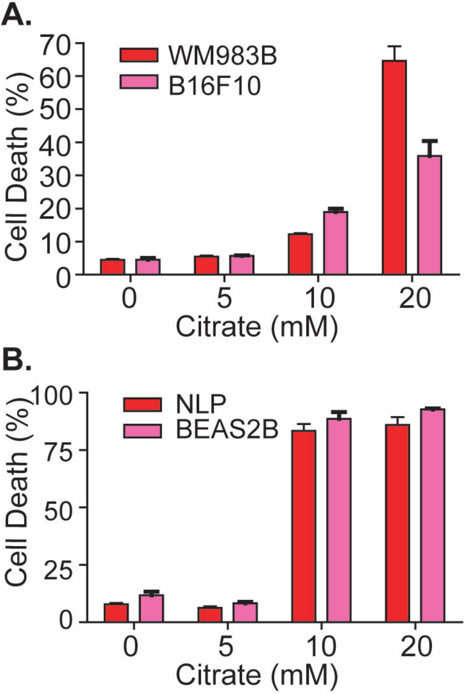

A.
WM983B
B16F10
Cell Death (%)
70
60
50
40
30
20
10
0
0
5
10
20
Citrate (mM)
B.
NLP
BEAS2B
Cell Death (%)
100
75
50
25
0
0
5
10
20
Citrate (mM)

Figure S3

A.

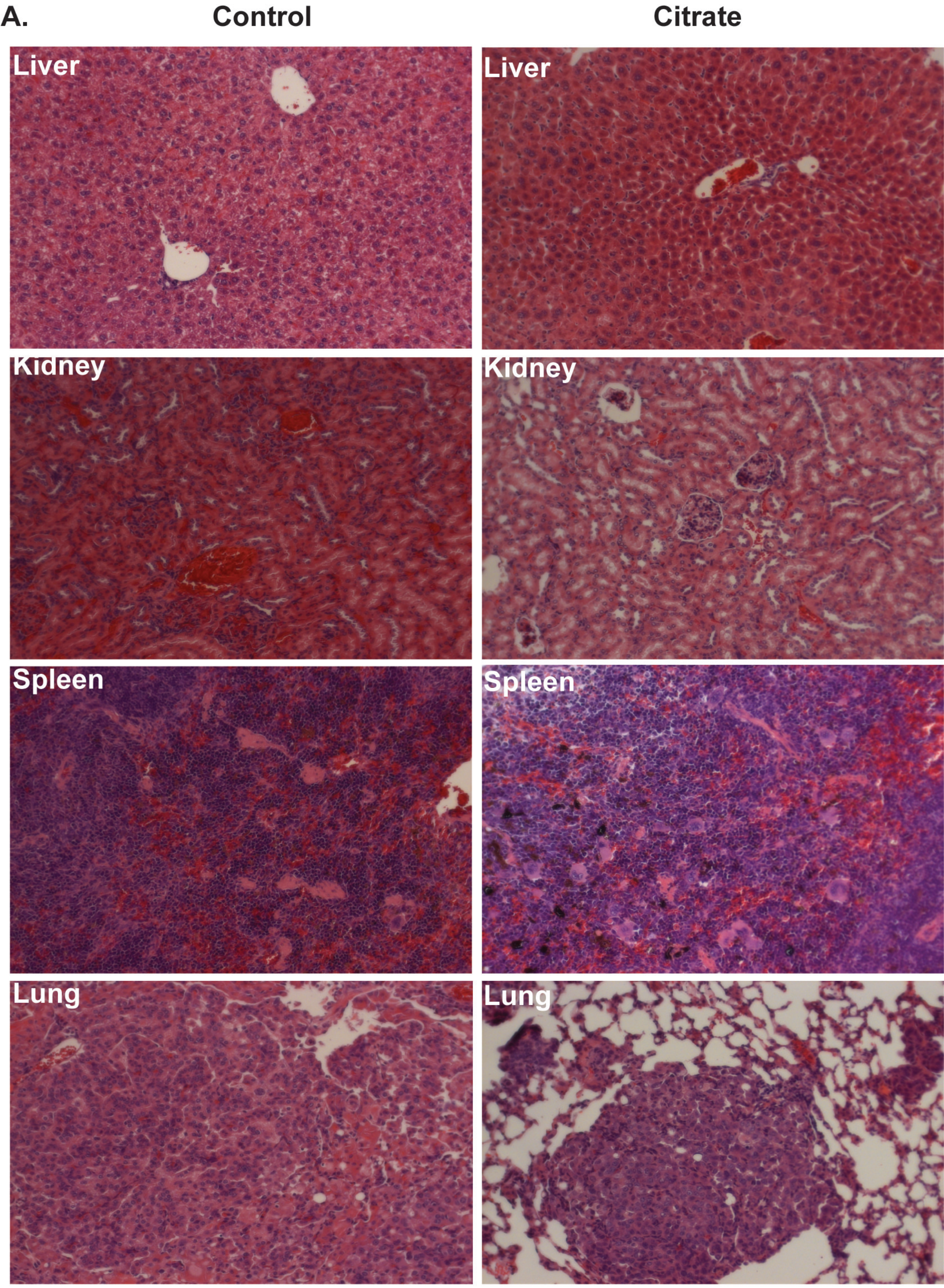

Control
Citrate
Liver
Liver
Kidney
Kidney
Spleen
Spleen
Lung
Lung

B.

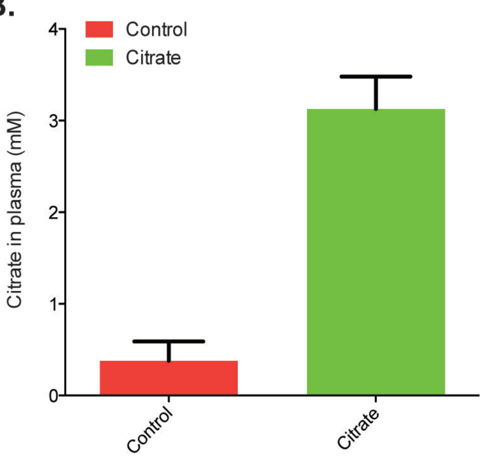

Control
Citrate
Citrate in plasma (mM)
0
1
2
3
4
Control
Citrate

Figure S4

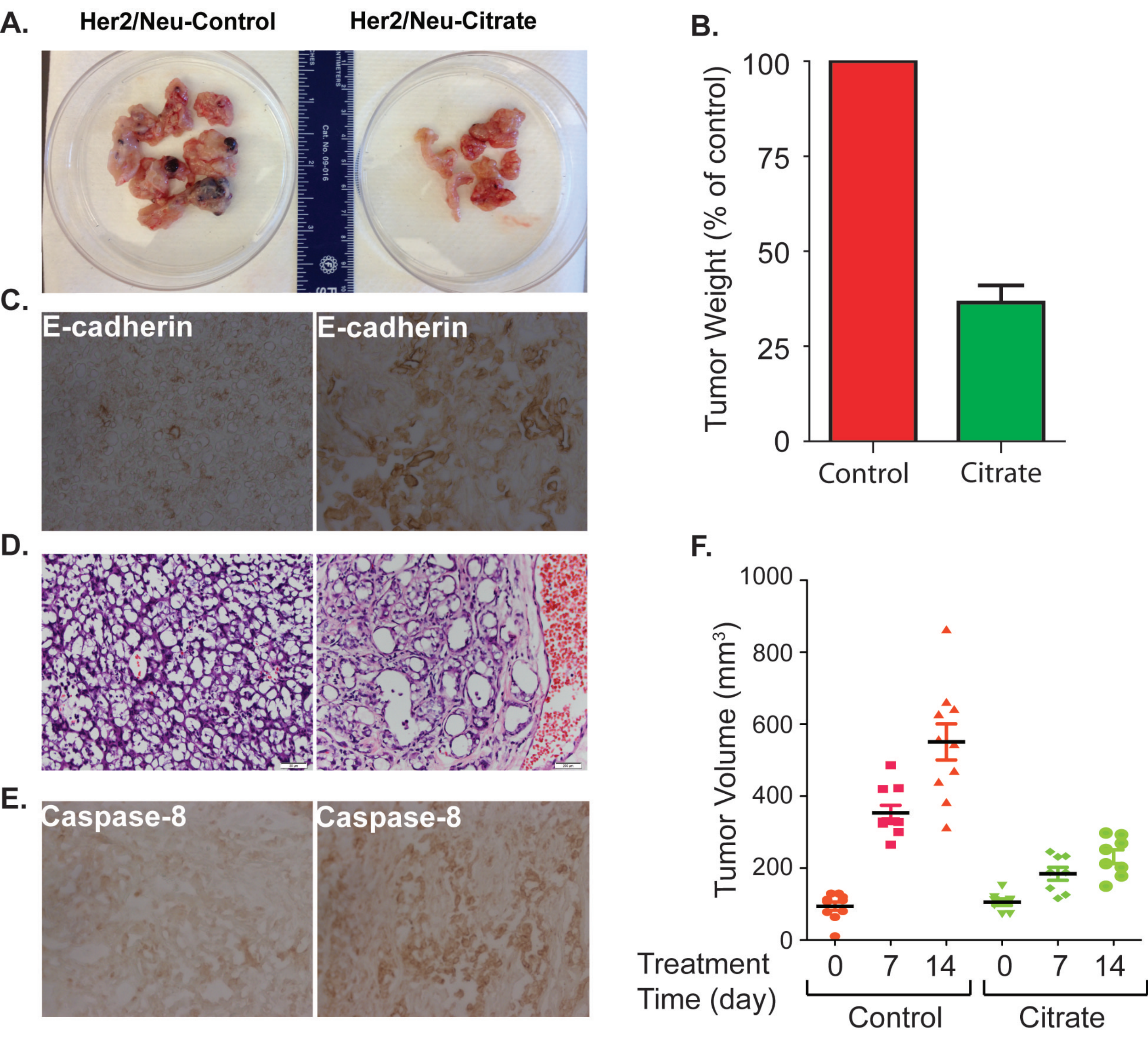
A.
Her2/Neu-Control
Her2/Neu-Citrate
B.
Tumor Weight (% of control)
100
75
50
25
0
Control
Citrate
C.
E-cadherin
E-cadherin
D.
E.
Caspase-8
Caspase-8
F.
Tumor Volume (mm³)
1000
800
600
400
200
0
Treatment Time (day)
0
7
14
0
7
14
Control
Citrate

Figure S5

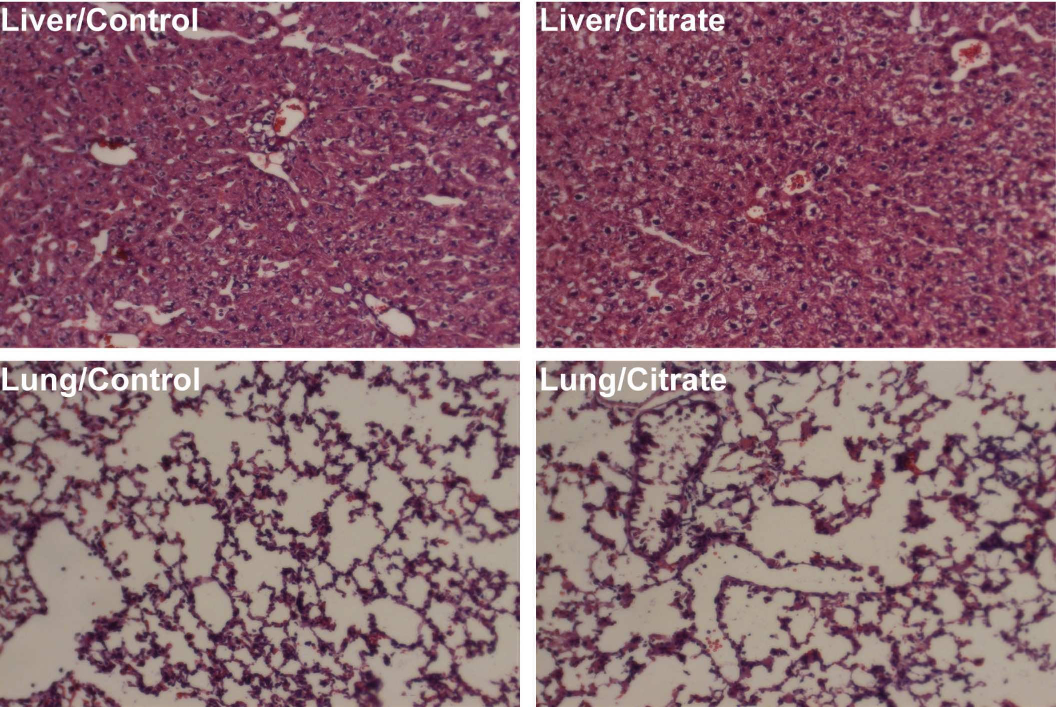

Figure S6

Her2/Neu

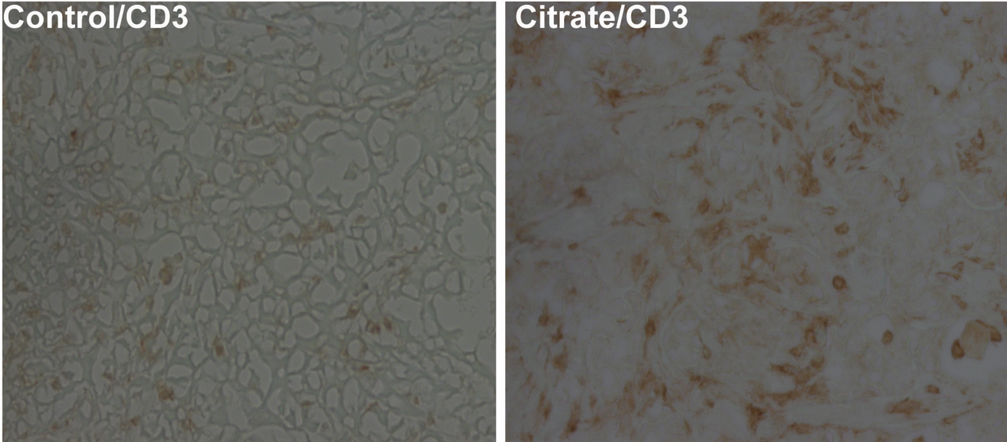

Figure S7

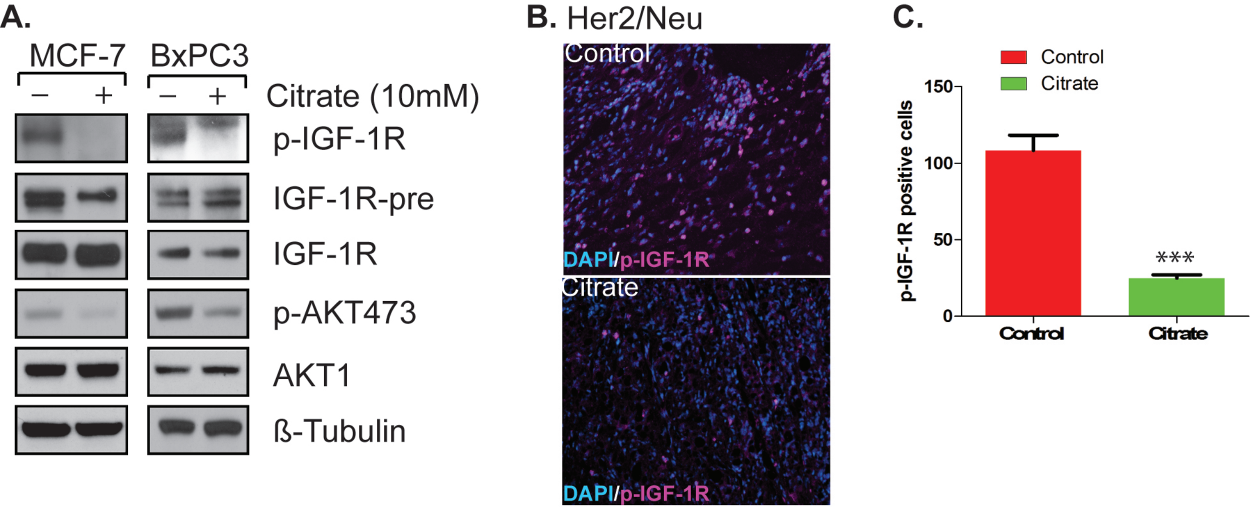
A.
MCF-7
BxPC3
– + – +
Citrate (10mM)
p-IGF-1R
IGF-1R-pre
IGF-1R
p-AKT473
AKT1
ß-Tubulin
B. Her2/Neu
Control
DAPI/p-IGF-1R
Citrate
DAPI/p-IGF-1R
C.
Control
Citrate
p-IGF-1R positive cells
150
100
50
0
***
Control
Citrate

Figure S8

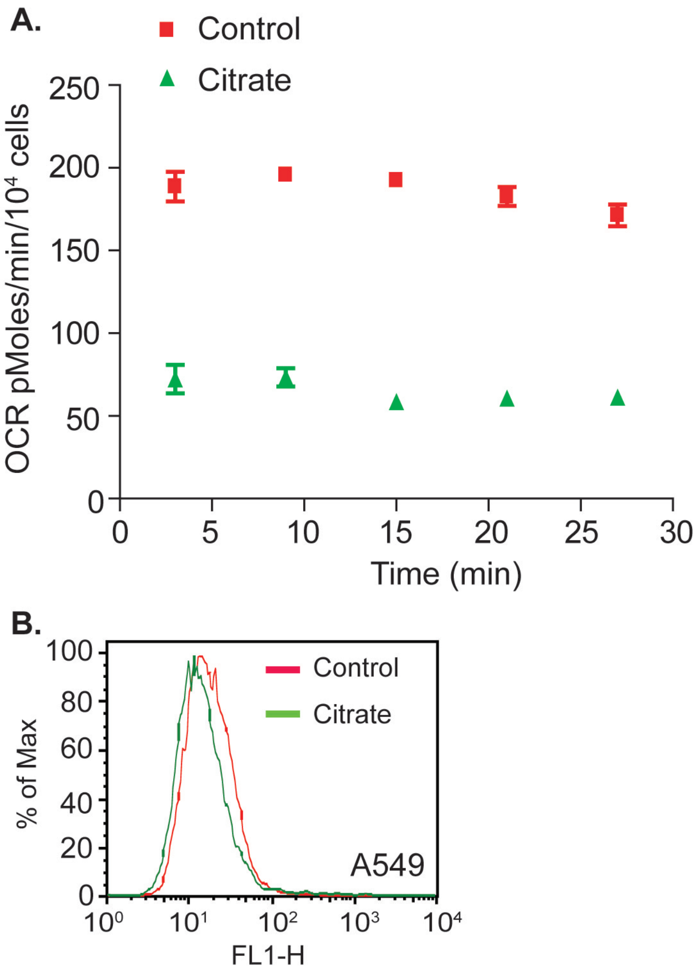
A.
Control
Citrate
OCR pMoles/min/10⁴ cells
250
200
150
100
50
0
0
5
10
15
20
25
30
Time (min)
B.
100
80
60
40
20
0
% of Max
Control
Citrate
A549
10⁰
10¹
10²
10³
10⁴
FL1-H

Figure S9.

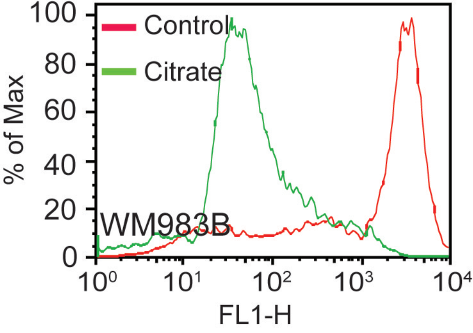

Control
Citrate
WM983B
100
80
60
40
20
0
% of Max
10^0
10^1
10^2
10^3
10^4
FL1-H

Figure S10

A. Her2/Neu Glycolysis

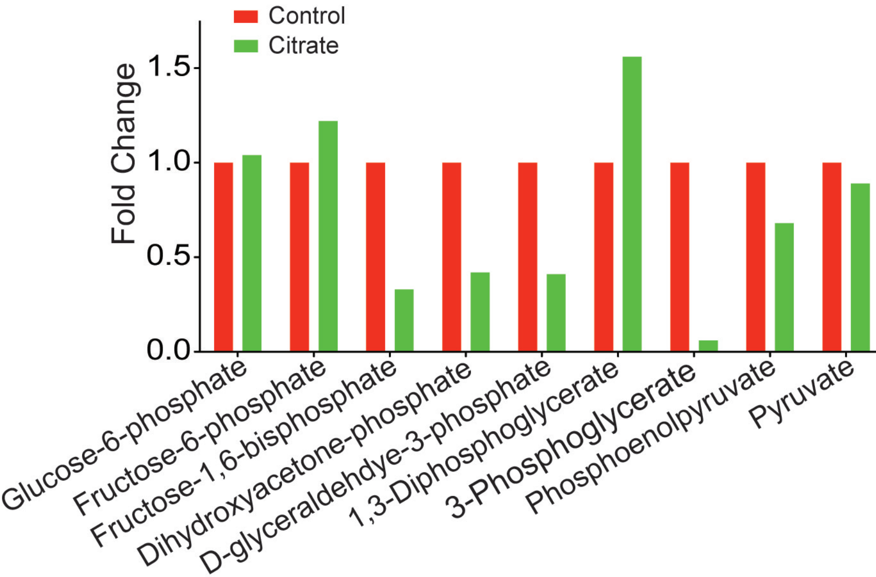

B. Her2/Neu TCA

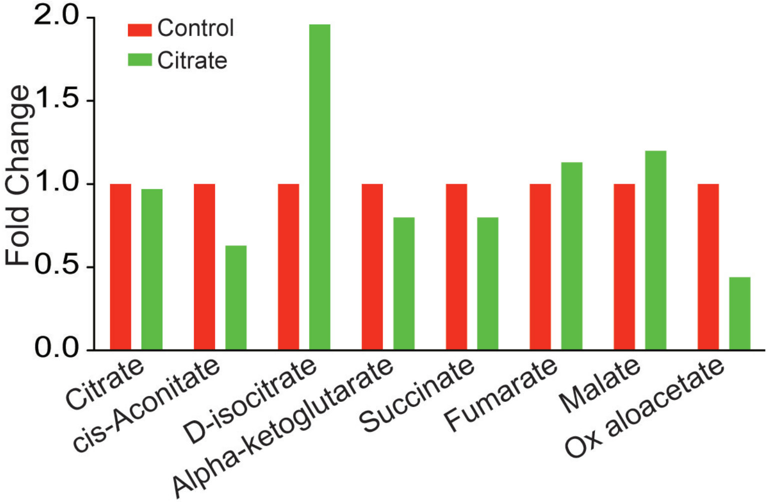

Figure S11

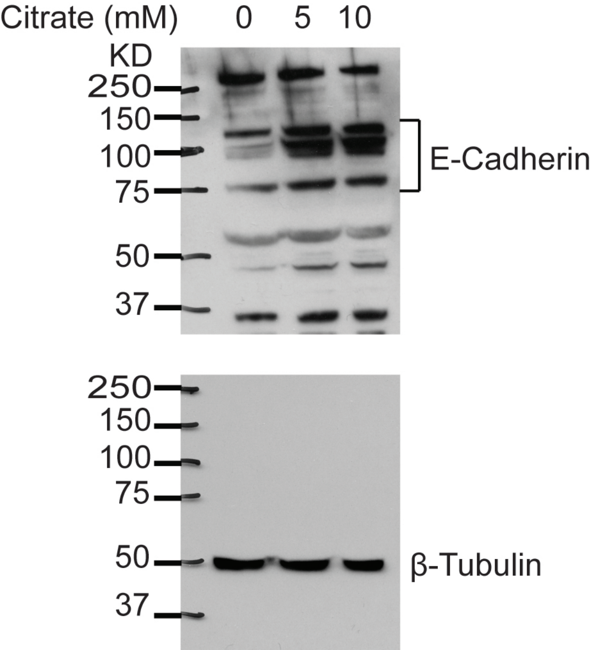

Citrate (mM) 0 5 10
KD
250
150
100
75
50
37
E-Cadherin
250
150
100
75
50
37
β-Tubulin

Figure S12

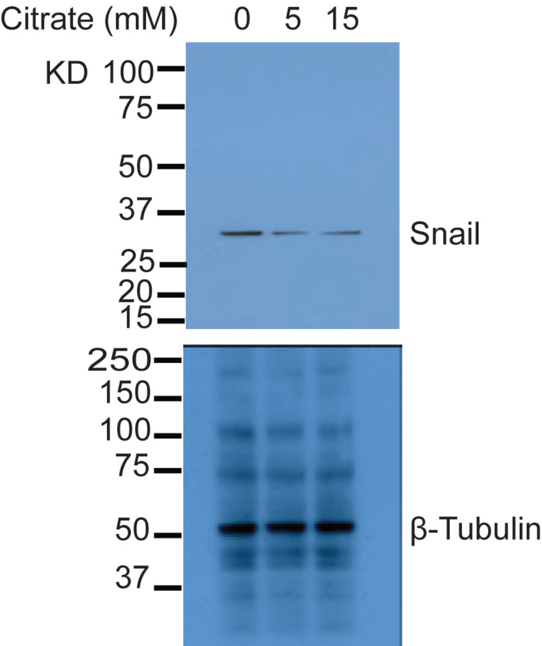
Citrate (mM) 0 5 15
KD 100
75
50
37
25
20
15
Snail
250
150
100
75
50
37
β-Tubulin

Figure S13

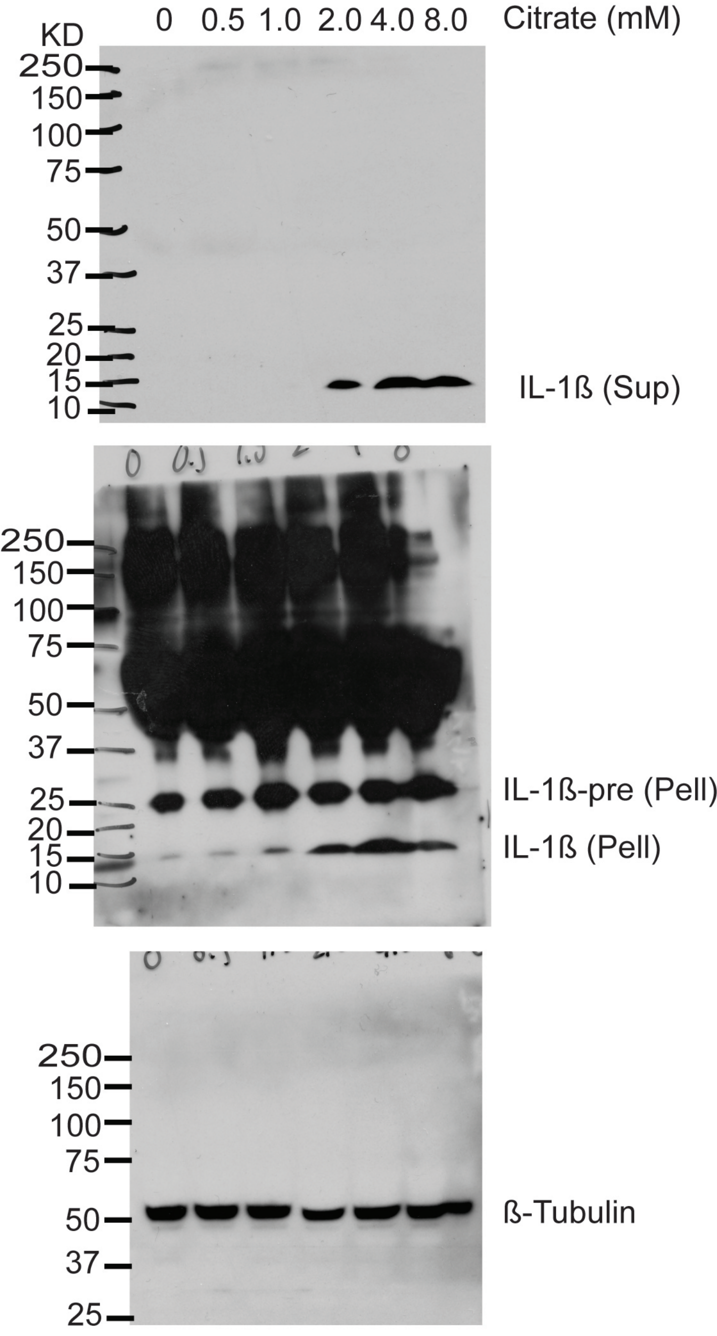
0 0.5 1.0 2.0 4.0 8.0 Citrate (mM)
KD
250
150
100
75
50
37
25
20
15
10
IL-1ß (Sup)
IL-1ß-pre (Pell)
IL-1ß (Pell)
ß-Tubulin

Figure S14

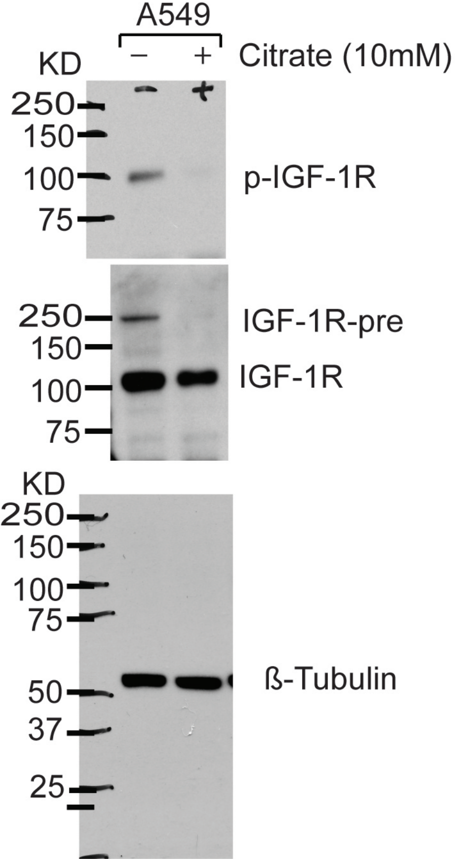
A549
–
+
Citrate (10mM)
KD
250
150
100
75
p-IGF-1R
250
150
100
75
IGF-1R-pre
IGF-1R
KD
250
150
100
75
50
37
25
ß-Tubulin

Figure S15

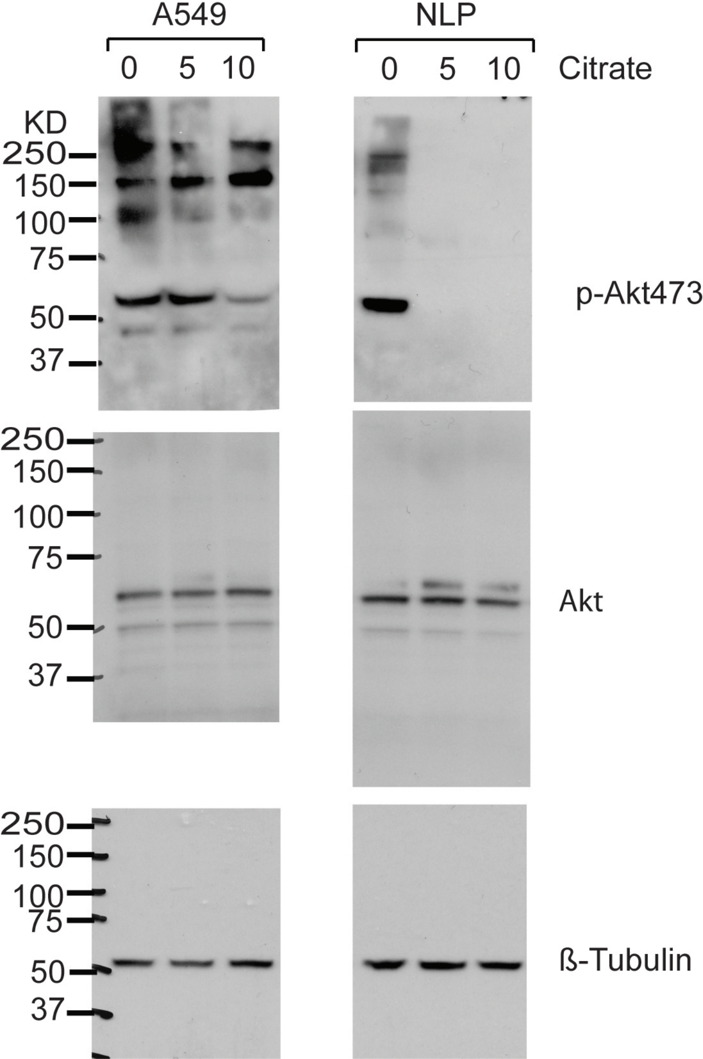
A549
NLP
0 5 10
0 5 10
Citrate
KD
250
150
100
75
50
37
p-Akt473
250
150
100
75
50
37
Akt
250
150
100
75
50
37
ß-Tubulin

Figure S16

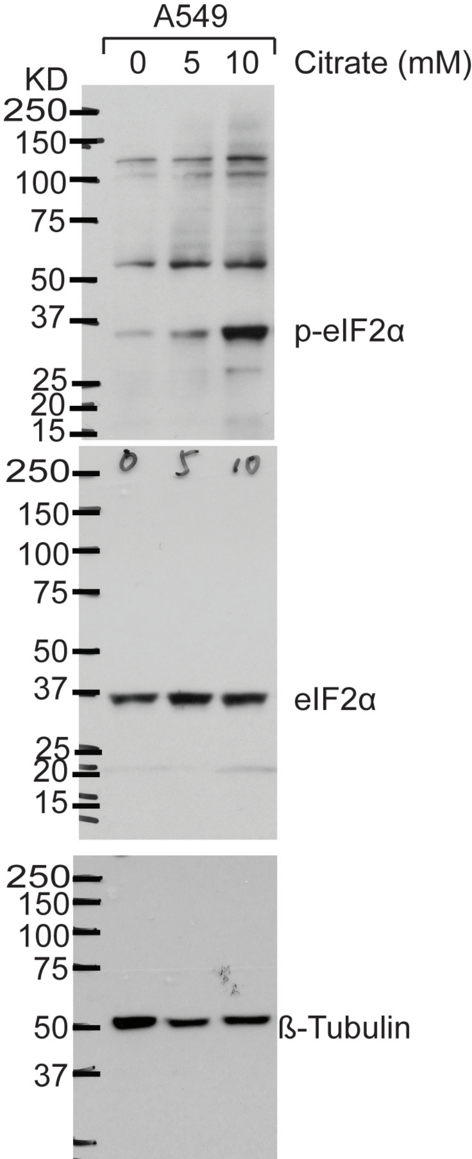

A549
0 5 10 Citrate (mM)
KD
250
150
100
75
50
37
25
20
15
p-eIF2α
0 5 10
eIF2α
ß-Tubulin

Figure S17

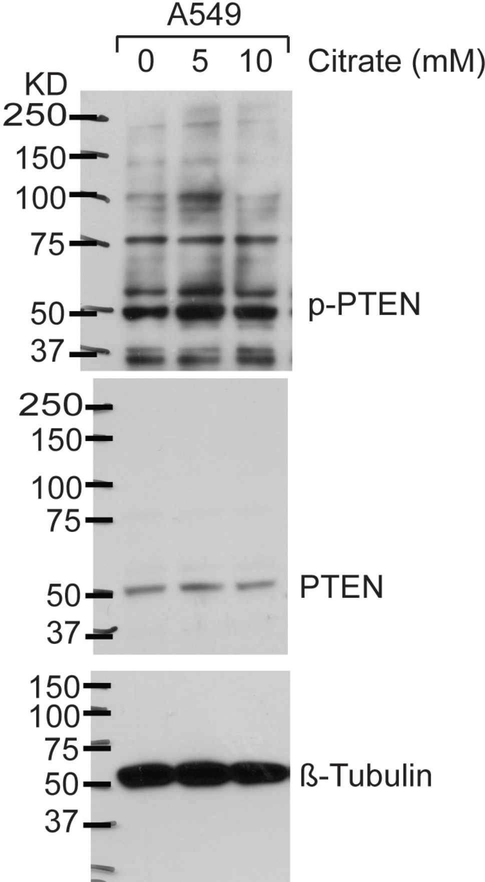
A549
0
5
10
Citrate (mM)
KD
250
150
100
75
50
37
p-PTEN
250
150
100
75
50
37
PTEN
150
100
75
50
37
ß-Tubulin

Figure S18

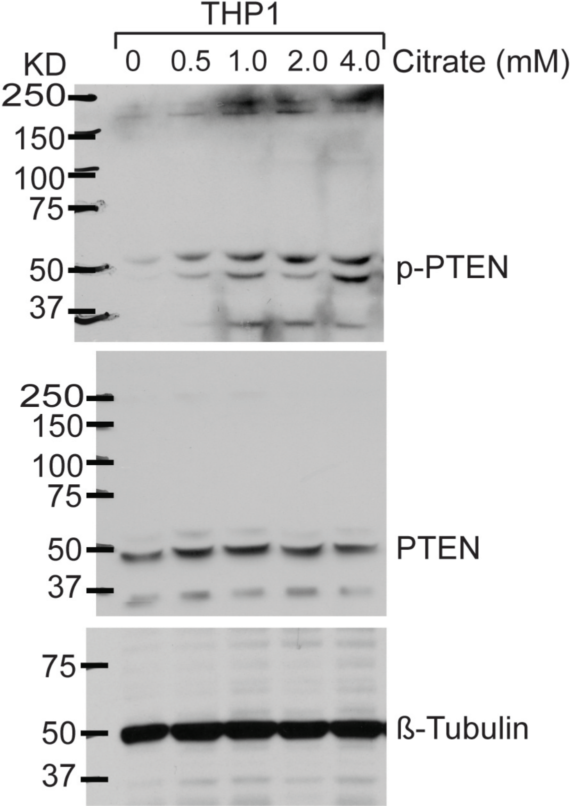
THP1
KD
0
0.5
1.0
2.0
4.0
Citrate (mM)
250
150
100
75
50
37
p-PTEN
250
150
100
75
50
37
PTEN
75
50
37
ß-Tubulin

Figure S19

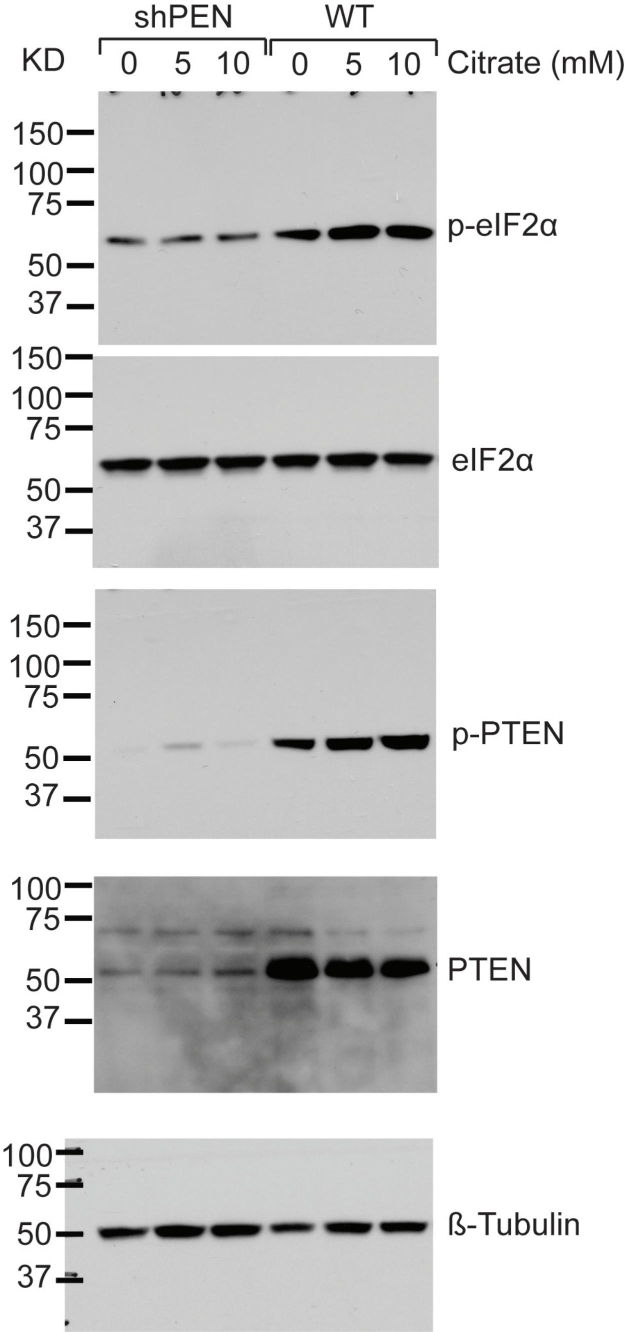
shPEN
WT
KD
0
5
10
0
5
10
Citrate (mM)
150
100
75
50
37
p-eIF2α
150
100
75
50
37
eIF2α
150
100
75
50
37
p-PTEN
100
75
50
37
PTEN
100
75
50
37
ß-Tubulin
